# Salinity dynamics in the Sundarbans of Bangladesh: influence of climate, freshwater inflow, and sea level changes

**DOI:** 10.1007/s10661-025-14667-2

**Published:** 2025-10-20

**Authors:** Shahriar Wahid, Mohammed Mainuddin, Francis Chiew, Fazlul Karim, Shaikh Nahiduzzaman, Rubayat Alam, Md. Raqubul Hasib

**Affiliations:** 1 Commonwealth Scientific and Industrial Research Organisation (CSIRO), Canberra, Australia; 2 Institute of Water Modelling, Dhaka, Bangladesh

**Keywords:** Sundarbans, Mangrove ecosystem, Coastal salinisation, Freshwater inflow, Climate change

## Abstract

The Sundarbans mangrove ecosystem, the world’s largest contiguous mangrove forest, is under pressure from changing salinity due to climate change, reduced freshwater input from upstream rivers, and tidal dynamics in the Bay of Bengal. Despite its ecological and socio-economic significance, previous studies have not provided a comprehensive, location-specific assessment of salinity that explicitly links spatial heterogeneity with hydroclimatic drivers. This study synthesised and analysed the best available data in the Bangladesh Sundarbans over the 26 years to quantify the characteristics, trends, and interrelationships of these variables spatially and temporally. The results reveal that salinity has been consistently higher in the western part of the Sundarbans, and extreme salinity levels have increased since 2010. There has been a statistically significant decrease in the freshwater inflow from the Ganges through the Gorai River (at the Gorai railway bridge location) since the early 1980s, which has led to increased downstream salinity. Strong statistical correlations enabled robust predictions of salinity as a function of rainfall and temperature. A rising water level trend at Hiron Point is attributed to the rise in sea levels. The absence of long-term measurement data prevents the exploration of salinity dynamics in the numerous creeks that crisscross the mangrove ecosystem. It is crucial to establish continuous monitoring and assessment to better understand and manage the impacts of climate change and development on the Bangladesh Sundarbans and similar coastal ecosystems worldwide.

**Graphical abstract:**

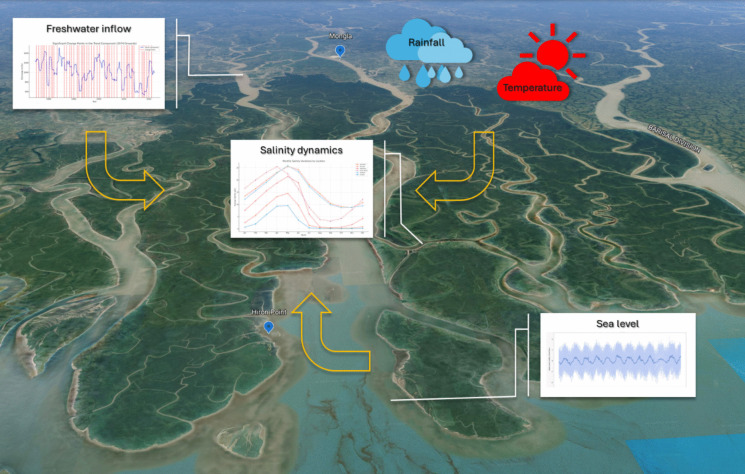

**Supplementary Information:**

The online version contains supplementary material available at 10.1007/s10661-025-14667-2.

## Introduction

The Sundarbans are a vast mangrove forest in the delta of the Ganges–Brahmaputra River system (Aziz & Paul, 2015; Sarkar & Chander, 2002). It is the world’s largest continuous mangrove forest, covering around 6000 km^2^ in Bangladesh and 4000 km^2^ in India (Gopal & Chauhan, 2006). In Bangladesh, the Sundarbans account for about 51% of the country’s total reserved forest area and are a region of transition between the freshwater of rivers originating from the Ganges and the saline water of the Bay of Bengal (Wahid et al., 2007). This unique ecosystem is home to a diverse array of plant and animal species, comprising 300 types of plants and 1760 animal species. The Sundarbans also hold significant cultural and economic importance, supporting the livelihoods of 10 million coastal residents who rely on it for agriculture, fishing, and forestry-related employment. It provides several ecosystem services such as provisioning services (e.g. forest products, food), cultural services (e.g. tourism, worship, educational research), regulatory services (e.g. protection from cyclones and storm surges, climate regulation, pollination), and supporting services (e.g. nursery ground of fish, nutrient cycling, habitat of biodiversity). The United Nations Educational, Scientific and Cultural Organisation (UNESCO) recognises three sanctuaries within the Sundarbans as World Heritage Sites.

Various factors, including climate change, rise in sea level, and salinity intrusion, threaten the Sundarbans ecosystem and its exceptional biodiversity and valuable ecological services (Dasgupta et al., 2017). The Sundarbans have experienced considerable degradation (Rahman & Rahaman, 2018). About 8507 ha of mangrove, essential in maintaining biodiversity and ecological balance, has been lost in the past 35 years (Zhang et al., 2023). Changes in climate and salinisation have caused significant losses in *Heritiera fomes* and substantial gains in *Excoecaria agallocha* in the Bangladesh Sundarbans, impacting the mangrove species in the region (Dasgupta et al., 2017). Climate change and sea level rise are projected to lead to significant habitat loss for the Bengal tiger in the Bangladesh Sundarbans (Mukul et al., 2019). Therefore, the urgency of understanding salinity dynamics (Islam & Gnauck, 2011), including the influence of upstream freshwater input, long-term rainfall change, and seawater levels, cannot be overstated. Understanding the changes to salinity dynamics is essential and crucial for assessing and mitigating the risk to the Sundarbans ecosystem. Even minor changes in salinity could render low-lying mangrove systems, such as the Sundarbans, vulnerable. Elevated salinity levels can induce osmotic stress in mangrove plants, disrupting their ability to absorb water and nutrients from the soil efficiently (Flowers & Colmer, 2008). This physiological stress can manifest as reduced growth rates, decreased photosynthetic activity, and, in extreme cases, mortality, particularly among less tolerant species (Ball, 1988).

Past studies have characterised the spatial and temporal variability in salinity in the Sundarbans rivers (Haq et al., 2024; Rahman & Rahaman, 2018; Trivedi et al., 2016). Salinity concentrations are generally highest in the dry season and lowest during the monsoon. Salinity generally increases almost linearly from October (post-monsoon) to late May (pre-monsoon). At the end of May, the salinity level drops sharply due to rainfall and increased upstream freshwater river flow, and it remains low until early October. In the dry season, when rainfall is minimal, the upstream freshwater inflow relies solely on flows through the Ganges River. In the monsoon (July–October), rainfall significantly boosts river discharge and reduces salinity levels (Anwar & Takewaka, 2014; Rahaman et al., 2015). Typically, salinity levels are higher closer to the coast and lower further inland.

Several studies have reported an increasing salinity trend across the Sundarbans in Bangladesh and India (Chowdhury et al., 2023; Islam & Gnauck, 2011; Zaman et al., 2013). Global climate change, alterations in regional hydrology, developmental pressure around the forests, and shifts in local socio-economic conditions are the primary factors contributing to the increase in salinity levels (Broucke et al., 2020). Reduction of freshwater flow from the Ganges River and its distributaries also contributes to the salinity increase in the Sundarbans’ coastal mangrove system (Akter et al., 2024a, b; Haq et al., 2024; Islam & Gnauck, 2011). The reduction in freshwater flow has been attributed to India’s construction of the Farakka Barrage, about 25 km upstream, before it enters Bangladesh (Islam & Gnauck, 2011; Mirza, 1998). The discharge from the Gorai River, the main distributary feeding the Sundarbans, has also declined since 1980 (Gain & Giupponi, 2014; Islam & Gnauck, 2008; Uddin & Haque, 2010). Attempts to dredge the Gorai Riverbed to enhance water flow during the dry season (Mohiuddin, 2002) had limited success (Rahman & Rahaman, 2018; Wahid et al., 2007). Increased saltwater intrusion due to sea level rise poses another significant challenge for the mangrove system (Bricheno et al., 2021). Significant seasonal variations in the salinity levels due to sea level rise in the Sundarbans have also been reported (Akter et al., 2024a, b; Chowdhury et al., 2021; Haq et al., 2024).

Although previous studies have investigated various aspects of mangrove hydrology, they do not provide location-specific analyses of salinity trends that explicitly link their observed spatial heterogeneity with the varying rates of hydroclimatic change across the monitoring network. Most studies identified a generalised seaward-landward salinity gradient. Still, they were limited in providing a granular, location-specific characterisation of spatial heterogeneity and understanding chronic spatial hotspots and emerging critical nuances in salinity distribution patterns. A comprehensive spatiotemporal assessment integrating salinity, climate, river discharge, and sea level rise, as well as a predictive salinity model based on climate, remains lacking. This investigation is of utmost importance, as it will enable the assessment of the extent of salinity variations in coastal environments, whether natural or induced by climate change, and enhance the understanding of the consequences of these changes on the resilience of the Sundarbans ecosystems.

This study addresses the gap by analysing and interpreting long-term climate, river discharge, sea level, and salinity datasets collected by the Bangladesh government agencies to provide insights into the salinity dynamics of the Sundarbans in Bangladesh. Here, we build on past studies and offer crucial insights to better understand the drivers of salinity in the Sundarbans, assessing how these have changed, and informing management and mitigation of the impact of changing salinity on the Sundarbans ecosystem. First, we hypothesised that the Sundarbans’ salinity has statistically significant seasonal and spatial gradients. To test this hypothesis, we analysed the long-term, seasonal, and spatial variations of salinity. Our second hypothesis is that a correlation exists between the seasonal and spatial salinity gradients and observed temperatures, rainfall, and upstream freshwater input. We assessed the impact of these drivers and developed predictive models of salinity based on their influences. Finally, we hypothesised that seawater level changes could provoke landward inundations and affect the frequency and intensity of salinity. We analysed salinity and water levels near the Bay of Bengal to investigate whether and how tidal levels contribute to river salinity.

## Materials and methods

### Study area

**Fig. 1:**
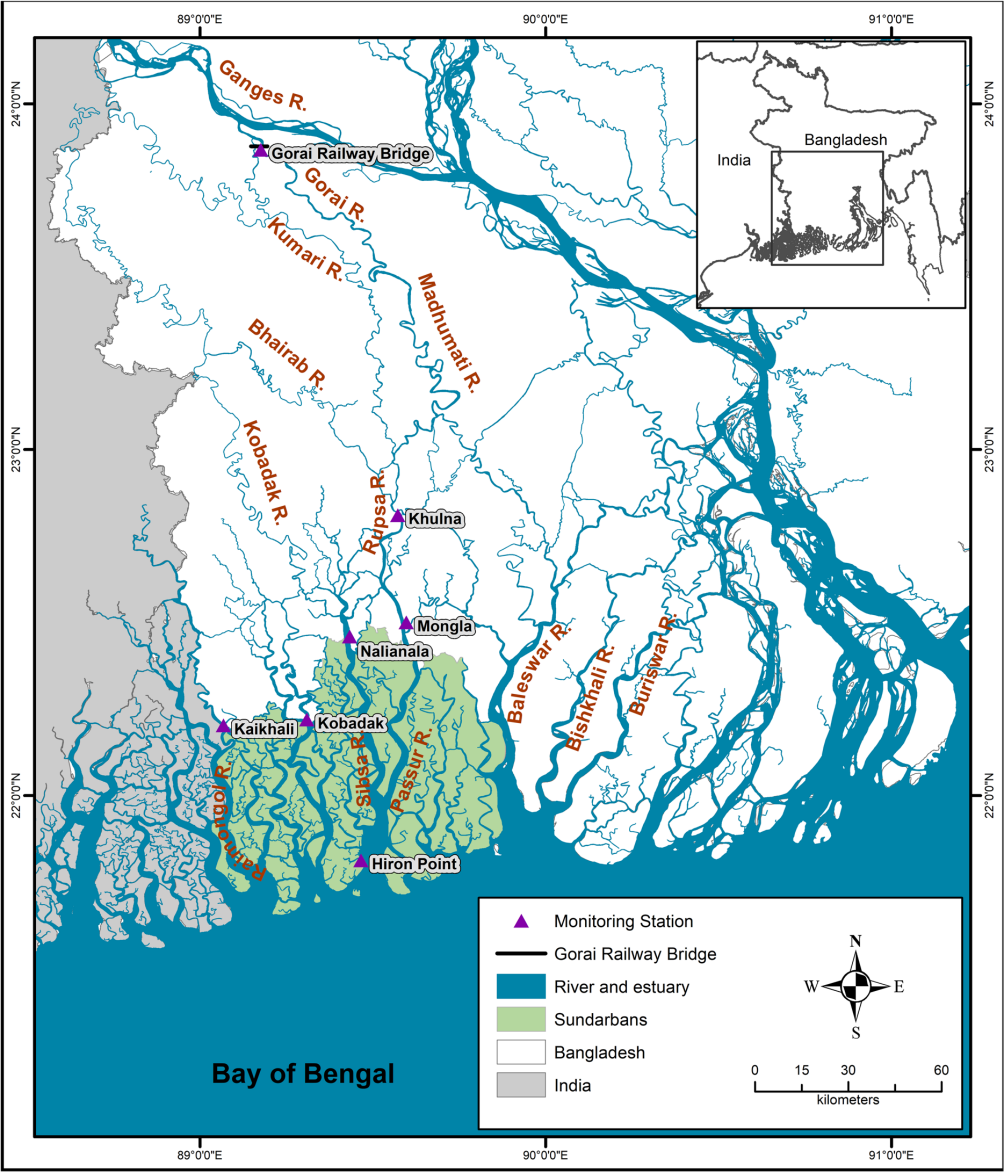
Monitoring stations’ data used in the study

The Sundarbans is on the mega delta formed by the transboundary Ganges, Brahmaputra, and Meghna (GBM) river network. Shared by southern Bangladesh and the southern part of West Bengal State of India, this is the largest single block of tidal halophytic mangrove forest in the world (Sarkar et al., 2019). The Sundarbans extend from 21°27′30″ to 22°30′30″ north latitude and 87°55′01″ to 89°00′00″ east longitude and cover an area of approximately 10,000 km^2^ (Fig. 1). Approximately 60% of the total Sundarbans lies in Bangladesh and 40% in India (Spalding et al., 2010). Unfortunately, acquiring consistent and long-term data from the Indian Sundarbans proved challenging within the scope of this study. Therefore, our analysis is limited to the Bangladesh Sundarbans, for which we compiled a robust dataset.

The unique morphological feature of the Sundarbans is a conglomeration of mudflats, coastal dunes, creeks, island beaches, estuaries, river channels, inlets, and mangrove swamps, where the individual components are interdependent. It comprises a large alluvial basin floored with Quaternary sediment deposited by the GBM rivers and their numerous distributaries (Ghosh et al., 2015; Sarkar et al., 2019). The Sundarbans present a complex ecosystem developed by an intricate system of tidal rivers, mudflats, and salt-tolerant mangrove forests (Hossain, 2018; Sarkar et al., 2019). With a tidal amplitude of more than 5 m, tidal influence extends to 130 km inland. This, combined with the low elevation of less than 3 m above mean sea level across a large region, has formed one of the world’s most extensive coastal lowlands (Snead, 2010).

The hydrology is dominated by freshwater flow from the Ganges, which exhibits very high seasonal variation. The Gorai River is the major distributary of the Ganges River and a significant source of freshwater to the channels and creeks of the Sundarbans. The Gorai River delivers freshwater to the Sundarbans’ mangrove forest through the Madhumati, Passur, and Sibsa rivers. These rivers are crucial to the health and sustainability of the Sundarbans’ ecosystems. On the eastern side, the Baleswor River forms the Sundarbans’ eastern boundary before emptying into the Bay of Bengal. The freshwater flows down the rivers with the tidal ingress, creating a gradient of salinity that varies spatially and temporally, with salinity being higher nearer the coast and lower further inland (Islam & Gnauck, 2011).

The region experiences a tropical climate with three distinct features throughout the year. The summer is hot and humid from March to June, with sporadic rainfall. This is followed by the monsoon from July to October. The winter, from November to February, is dry (Ghosh et al., 2015; Iftekhar & Islam, 2004). Annual precipitation ranges from 1500 to 2000 mm. Approximately three-quarters of the annual rainfall occurs during the monsoon months, resulting in high river discharge that gradually decreases for the remainder of the year. The timing and amount of monsoon rainfall have become increasingly variable in the delta (Chowdhury et al., 2016). During the summer and winter, the area is frequently impacted by tropical cyclones and tidal events, resulting in severe flooding and wind damage. Over time, the Sundarbans have weathered numerous cyclones, further shaping the area’s environmental landscape. Seasonal average temperatures vary, with minimums ranging from 12 °C to 24 °C and maximums from 25 °C to 35 °C (Ghosh et al., 2017).

Mangroves are the dominant geomorphic agent in the evolution of tidal shoals and their accretion to the mainland. Their presence is vital in shoreline stabilisation, reduction of coastal erosion, sediment and nutrient retention, storm protection, flood and flow control, and water quality (Giri et al., 2007; Islam & Gnauck, 2011). Mangroves also reduce wave energy by obstructing the waves with their roots and trunks. The basic unit of management of the Sundarbans is the “compartment”. There are 55 compartments in the four Forest Ranges (Hossain et al., 2016) in the Sundarbans, demarcated mainly by natural features such as rivers, canals, and creeks.

### Data

We collated and curated rainfall, temperature, water level, salinity, and discharge data from the Bangladesh Water Development Board (BWDB), Mongla Port Authority (MPA), Bangladesh Meteorological Department (BMD), and Institute of Water Modelling (IWM). Table 1 lists the data used in the study, and Fig. 1 shows the locations of the measurement stations.

**Table 1:** Hydrometeorological and salinity data used in the study

Data type	Station	River	Data length	Data frequency (hr)	Data source
Water level	Khulna	Rupsa	1/01/1996–30/06/2022	3	BWDB
	Mongla	Passur			
	Nalianala	Sibsa			
	Kobadak	Kobadak			
	Kaikhali	Ichamati			
	Hiron Point	Passur		0.5	MPA
Discharge	Gorai RB	Gorai	1/01/1996–30/06/2022	3	IWM
Salinity	Khulna	Rupsa	15/01/2009–30/06/2022	12	
	Mongla	Passur	9/05/1998–30/06/2022	12	
			2/01/2011–30/06/2022	0.5	
	Nalianala	Sibsa	1/12/2008–30/06/2022	12	
	Kobadak	Kobadak	25/11/2000–30/06/2022	6	
	Kaikhali	Ichamati	27/11/2000–30/06/2022	1	
	Hiron Point	Passur	2/01/2011–30/06/2022	0.5	
			3/05/1998–30/06/2022	2	
Rainfall, Temp (max, min)	Khulna		1/01/1998–31/12/2022	24	BMD
	Mongla				

The BWDB regularly monitors water levels in major rivers (including the six locations in Fig. 1 and Table 1) around the Sundarbans, and the MPA monitors the tidal level near the Bay of Bengal at Hiron Point. The IWM monitors salinity at the six locations in Table 1. The IWM measures the river discharge data at Gorai, which provides freshwater input into the Sundarbans. The rainfall and temperature data at two locations (Khulna and Mongla) come from the BMD. Salinity is measured using portable conductivity meters. The meters can measure 0–42 ppt and have a resolution of 0.01 ppt. They are calibrated at the IWM lab in Dhaka. Salinity is measured at the surface only, as vertical salinity stratification is subdued due to robust tidal mixing (Chatterjee et al., 2021).

These datasets are the most authoritative long-term time series data on climate, water level, river discharge, and salinity in Bangladesh and have been used in various studies on the Sundarbans. For example, the water level data of the Ganges Delta collected by the BWDB were used to assess the impact of salinity intrusion on agricultural productivity in coastal Bangladesh (Ashrafuzzaman et al., 2022). The salinity levels were correlated with crop yields to assess future risks under various climate scenarios. The study also used hydrologically modelled water levels and salinity intrusion from the Institute of Water Modelling (IWM). The IWM data were used to simulate scenarios related to river flow, salinity intrusion, and the impacts of various interventions, such as river dredging and the Ganges Barrage. Another study used time series records of surface water salinity and river discharge collected by the BWDB to analyse governance practices in managing salinity and climate change impacts in coastal regions (Haq et al., 2024).

The river discharge data from BWDB has been used to study the impact of water scarcity and salinity on agricultural practices (Khan et al., 2024). They utilised the Southwest Region Model (SWRM), maintained by IWM, to evaluate the impacts of water withdrawal and sea level rise on river salinity in the coastal regions of Bangladesh. A study by Jabir et al. (2021) examined the effects of climate change on aquatic ecosystems and fishery resources near the Sundarbans using daily maximum, minimum, and mean temperature records from the BMD. Others have also used the BMD rainfall and temperature records to investigate how climate change affects carbon accumulation in mangrove species (Chowdhury et al., 2024).

### Statistical analysis

#### Salinity variation and trends

Salinity variations were analysed by examining the distribution of daily salinity data (2009–2022) from six monitoring stations: Khulna, Mongla, Nalianala, Hiron Point, Kaikhali, and Kobadak using box plots. The median and interquartile range (IQR) were used to describe the central tendency and variability of salinity at each location. The median provided information on the typical salinity value, while the IQR represented the spread of the middle 50% of the data. Outliers were identified as data points falling beyond 1.5 times the IQR from the upper and lower quartiles.

A comprehensive trend analysis was conducted using high-resolution daily salinity data. A linear regression analysis was performed on daily salinity values to quantify the magnitude and direction of the detected trends over time. The slope of the regression line indicated the rate of change in salinity. At the same time, the significance of the model was assessed using one-way analysis of variance (ANOVA), with *F*-ratios and *p*-values serving as indicators of trend strength and reliability. We utilised daily salinity data to preserve the inherent variability of salinity in a tidal estuarine system, thereby providing a nuanced understanding of spatiotemporal salinity dynamics in the Sundarbans.

Seasonal variation was investigated using average salinity data (2009–2022) for three seasons: Summer (March to June), Monsoon (July to October), and Winter (November to February). This data aggregation allowed for a comparison of salinity patterns between seasons. We employed a two-way ANOVA to assess the effects of season, station, and their interaction on salinity concentrations, addressing four key questions: (1) Are there differences in salinity between seasons? (2) Are there differences in salinity between stations? (3) Are the differences between seasons the same at each station? and (4) Are differences between stations the same during each season? The model included season and station as fixed factors. Least squares means were calculated to estimate marginal means for main effects and interactions. A Tukey’s honestly significant difference (HSD) post hoc test was conducted to evaluate pairwise differences among the interactions. Effect screening using orthogonal estimates was conducted to identify the relative importance of factors.

#### Influence of climate and freshwater inflow on salinity

We evaluated the influence of climate on salinity at two locations, Khulna and Mongla, where we had robust datasets of climate and salinity (Fig. 1). We related salinity changes to concomitant variations in rainfall and temperature (*T*_max_ and *T*_min_) at these locations. We hypothesised that higher temperatures and lower rainfall would increase salinity levels. Long-term changes should interact with the spatial variations of salinity, with proportionally greater salinity closer to the Bay of Bengal. We acknowledge that evapotranspiration is an important factor in the water balance. However, it could not be included in the analysis due to a lack of a reliable long-term evapotranspiration dataset for the Sundarbans region. We expect that correlating temperature, rainfall, and discharge with salinity allows us to examine the combined influence of these key climatic factors on salinity dynamics. The time series plots of key climatic factors are provided in the Supplementary Materials.

The Gorai River, a distributary of the Ganges, plays a crucial role in moderating salinity in the Sundarbans. Reduced flow in the Gorai, often due to upstream water diversion and climate variability, exacerbates salinity levels, adversely affecting the delicate ecosystem of the Sundarbans mangrove forest (Aziz & Paul, 2015). Therefore, we evaluated how discharge at the Gorai Railway Bridge (RB) changed over time (1973–2022), influencing salinity levels at Mongla. We employed the Mann–Kendall test to assess temporal trends in discharge. The Mann–Kendall test is a non-parametric statistical test suitable for our analysis as it does not assume that the data are normally distributed and is relatively robust to outliers.

We used JMP software (JMP, Version 16.1; SAS Institute Inc., Cary, NC, 1989–2023) to quantify the correlation between salinity (dependent variable) and rainfall, temperature, and discharge, taking into account temporal lags. Monthly averages of salinity (ppt), rainfall (mm), and temperature (maximum and minimum in degrees celsius) were compiled from corresponding data for the 2009–2022 period. The cross-correlation function was selected due to its delayed salinity response to rainfall, temperature, and discharge variability, as well as its ability to identify optimal lags. The JMP uses the following steps to calculate the cross-correlations:

Each time series *X*_*t*_ and *Y*_*t*_ is first normalised by subtracting its mean and dividing by its standard deviation:

$${X}_{t}^{\prime}= \frac{{X}_{t}- \mu x}{\sigma x}, {Y}_{t}^{\prime}= \frac{{Y}_{t}- \mu y}{\sigma y}$$

where:

$\mu x and \mu y$ are the means of *X*_*t*_ and *Y*_*t*_

$\sigma x and \sigma y$ are the standard deviations of *X*_*t*_ and *Y*_*t*_

The cross-correlation function (CFF) *C*_*XY*_(*k*) at lag *k* is calculated as:

$${C}_{XY \left(k\right)}= \frac{1}{N-k} \sum\limits_{t=1}^{N-k}{X}_{t}^{\prime} {Y}_{t+k}^{\prime}$$

where:

*k* is the lag, which can be positive or negative

*N* is the length of the time series

${X}_{t}^{\prime}$ and ${Y}_{t+k}^{\prime}$ are the normalised values of the time series at time *t* and *t* + *k* respectively

${Y}_{t}^{\prime}$ represents the salinity time series (the response) and ${X}_{t}^{\prime}$ represents the rainfall, temperature, or flow time series (the covariate series). The calculated cross-correlation values ${C}_{XY \left(k\right)}$ for different lags, *k*, are plotted to visualise the relationship between the time series over various lags.

Using the results of the cross-correlation analyses, a multiple linear regression approach was implemented to model the relationship between salinity (dependent variable) and climatic factors with multiple temporal lags. The general form of the model can be expressed as:

$${\text{Salinity}}_{t}= {\beta }_{0} +\sum_{i=1}^{3}{\beta }_{i}{ \text{Rainfall}}_{t-i}+ {\beta }_{4}{ T\text{max}}_{t}+ {\beta }_{5}{ T\text{min}}_{t}+ {\varepsilon }_{t}$$

where:

${\text{Salinity}}_{t}$ represents average monthly salinity at time *t* (in ppt)

${\text{Rainfall}}_{t-i}$ denotes average monthly rainfall with lags of *i* = 1, 2, and 3 months

${T\text{max}}_{t}$ and ${T\text{min}}_{t}$ represents average monthly maximum and minimum temperatures

${\beta }_{0}$ through ${\beta }_{0}$ are regression coefficients

${\varepsilon }_{t}$ is the error term

The model fitting and diagnostics were done using a significance threshold of 0.05. Model performance was evaluated using multiple criteria, including the coefficient of determination (*R*^2^) and root mean squared error (RMSE).

The relationships between river discharge at Gorai RB location and salinity at Mongla and Hiron Point were analysed separately for the monsoon, summer, and winter seasons. The discharge–salinity relationships are complex and non-linear (Qiu & Wan, 2013). Conventional linear regression methods are inadequate for capturing the true nature of these relationships, especially when thresholds or seasonal shifts affect salinity dynamics. To address this limitation and effectively evaluate the strength and direction of the association, we used a non-parametric measure (Spearman’s *ρ*) that does not assume a specific functional form and is less sensitive to outliers and non-linear trends. Spearman’s *ρ* is based on ranks rather than the original values and ranges from −1 to +1, with larger absolute values indicating a stronger relationship. This approach allowed us to quantify monotonic relationships between discharge and salinity across different seasons, thereby revealing key seasonal variations that linear methods might miss.

#### Influence of sea level on salinity

We analysed tidal water levels at Hiron Point near the Bay of Bengal to investigate whether and how variations and changes in the sea level contribute to salinisation through local inundations. We hypothesised that higher sea levels would provoke landward inundations, which may have increased in frequency and intensity under climate change.

The time series decomposition technique in JMP was used to analyse the relationship between salinity and water level time series, including the underlying trends and seasonal patterns. The technique extracts the components in the time series to estimate the trend and seasonality and characterises the residual or random noise. Depending on the nature of the data, the decomposition can be performed using either an additive or multiplicative model. We used the additive model, where the components are added together, assuming that the seasonal fluctuations are roughly constant.

1 $$Y_t=T_t+S_t+E_t$$

where:

${Y}_{t}$ is the observed value at time *t*

${T}_{t}$ is the trend component at time *t*

${S}_{t}$ is the seasonal component at time *t*

${E}_{t}$ is the residual or error component at time *t*

The time series decomposition method used here requires continuous data. For this study, gaps in the data were filled using linear interpolation. We also resampled the original data, measured at different times, to obtain concurrent daily time series for the salinity and water level data.

## Results

### Salinity variation and trends

We analysed the salinity data from the six locations from 2009 to 2022 to quantify the spatiotemporal trends and variations. The data was smoothed using a rolling mean with a 24-h window to account for daily fluctuations in tidal areas. Figure 1 shows the six salinity measurement locations of the Sundarbans: Khulna, Mongla, Nalianala, Kobadak, Kaikhali, and Hiron Point.

**Fig. 2:**
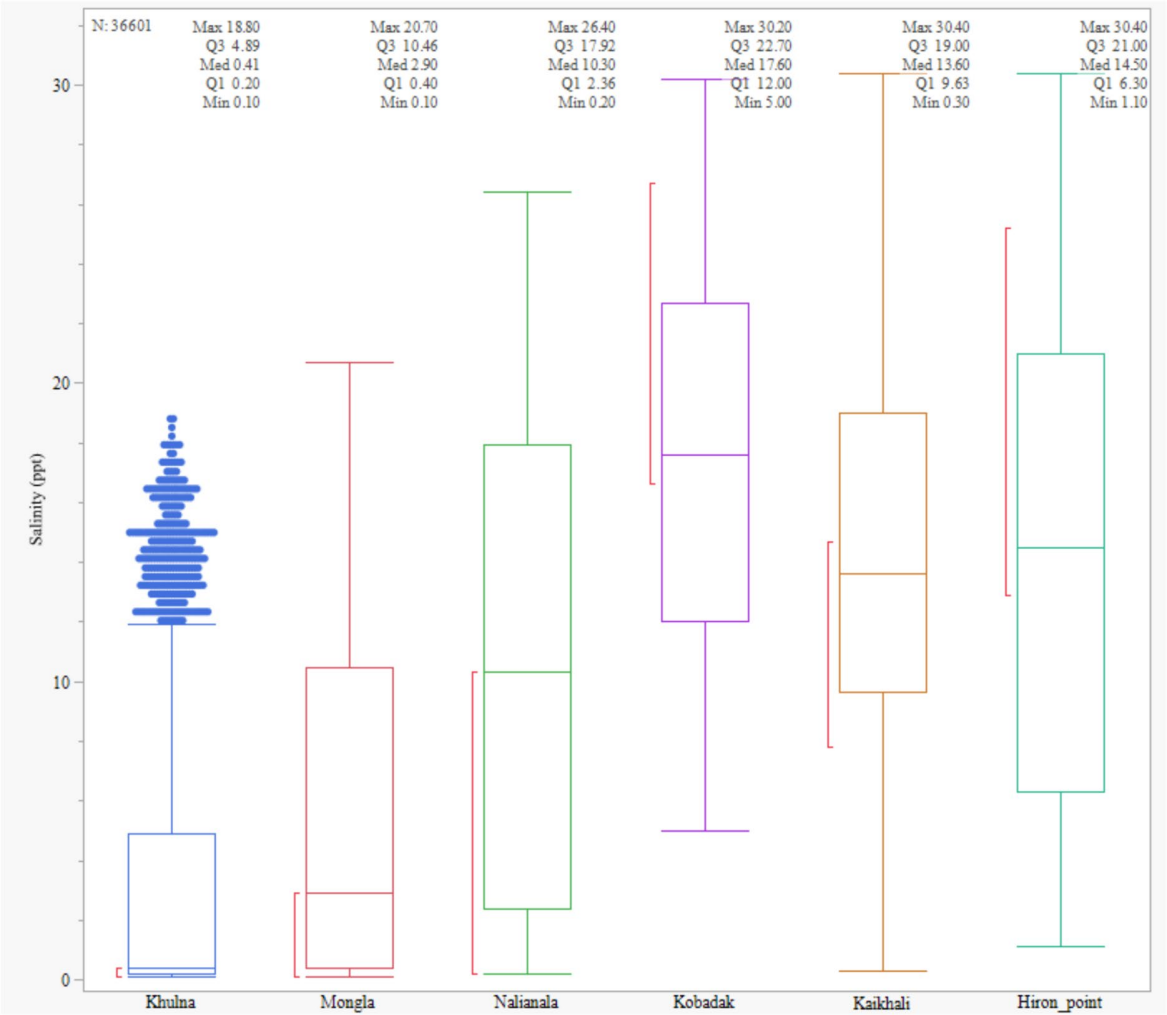
Salinity variation at monitoring stations. This figure presents differences in median, interquartile range, and outlier frequency, highlighting the spatial heterogeneity of salinity (ppt) in the Sundarbans. The stations are ordered based on their distance from the seafront, with Khulna furthest away

Box plots were prepared to compare salinity variation across different stations (Fig. 2). The central line in each box represents the median salinity value for each station. The median shows the central tendency of salinity measurements at each location. The height of each box indicates the interquartile range, representing the middle 50% of the data (from the 25th to the 75th percentiles). A smaller box height suggests that the salinity values are closer to the median, while a taller box indicates more variability. Points outside the “whiskers” (the lines extending from the top and bottom of each box) are considered outliers and show salinity measurements significantly higher or lower than the rest of the data at that station. Some stations have higher median salinity, while others may show more variability (wider boxes). The presence of outliers suggests occasional extreme salinity levels at Khulna station.

Salinity levels are consistently low at Khulna, with a median value of approximately 0.41 ppt (Fig. 2). The data shows a narrow interquartile range (IQR), indicating low variability in salinity across the years. This low variability suggests that Khulna experiences stable freshwater conditions, potentially due to effective river discharge during most seasons. Salinity levels at Kobadak exhibit a more comprehensive range, with a median value of around 17.6 ppt. The upper quartile reaches as high as 22.7 ppt, indicating higher salinity during specific periods, possibly in the dry season or when freshwater inflow is reduced. Kaikhali’s salinity distribution shows a similar pattern to Kobadak, with a slightly lower median value of around 13.6 ppt. The broader range of salinity levels suggests that Kaikhali experiences significant seasonal variations. Salinity at Nalianala has a median of 10.3 ppt, with the data showing a relatively consistent range. The station exhibits moderate variability, indicating a balance between freshwater inflow and saline water intrusion over the years. Mongla exhibits lower salinity levels than Kobadak and Kaikhali, with a median value of around 2.9 ppt. The narrower IQR suggests that salinity here is more stable but still shows some seasonal variation. Kobadak displays the highest salinity levels, with a median of around 17.6 ppt and maximum values reaching up to 30.2 ppt. The wide range and high quartiles indicate significant seasonal variation, with possible extreme salinity during dry periods.

**Fig. 3:**
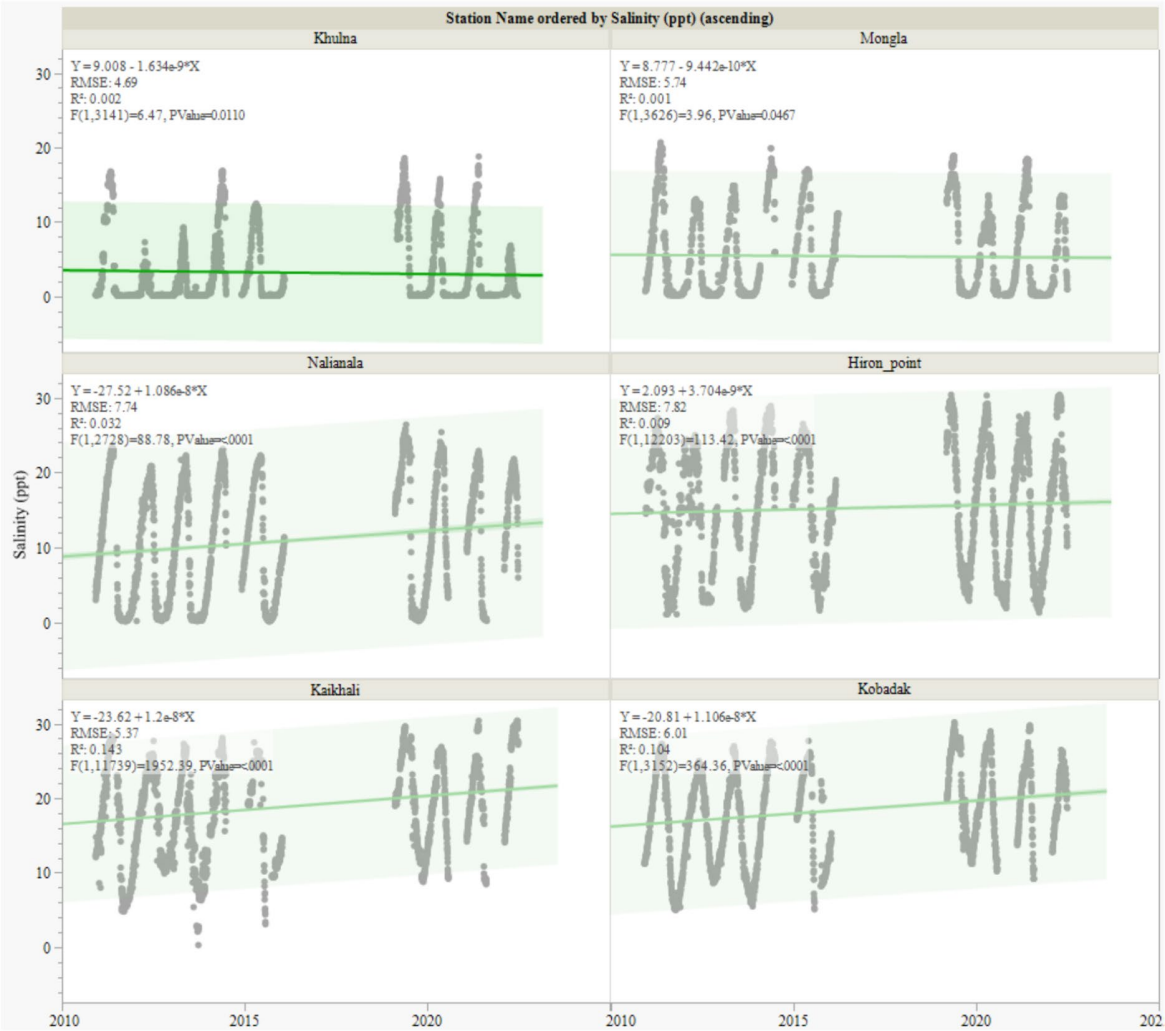
Temporal trends in salinity (ppt) at six monitoring stations in the Sundarbans region: Khulna, Mongla, Nalianala, Hiron Point, Kaikhali, and Kobadak. Each plot shows the salinity measurements (grey points) over time, with a fitted regression line (green) indicating the direction and magnitude of the trend. The accompanying Analysis of variance (ANOVA) provides the sum of squares, mean square, *F*-ratio, and *p*-value, assessing the statistical significance of the trends

A linear regression analysis was conducted to investigate the temporal dynamics of salinity across six monitoring stations: Khulna, Mongla, Nalianala, Hiron Point, Kaikhali, and Kobadak. Each station’s salinity data was regressed against time to reveal the trends (Fig. 3).

At Khulna, the regression model indicated a statistically significant negative trend in salinity over time (*F* = 6.46, *p* = 0.011), reflecting a modest but detectable decrease. Mongla also exhibited a negative trend (*F* = 3.95, *p* = 0.0467), albeit with a smaller effect size, implying a subtle upward shift in salinity levels. In contrast, Nalianala demonstrated a significant increasing trend (*F* = 88.78, *p* < 0.0001), characterised by a steep slope indicative of pronounced salinity escalation, potentially attributable to local environmental changes. Hiron Point also presented a significant upward trend in salinity (*F* = 113.41, *p* < 0.0001), suggesting a persistent salinisation process possibly linked to diminished freshwater inflows or heightened tidal influence. Kaikhali exhibited one of the steepest slopes among the stations, with a highly significant trend (*F* = 1952.38, *p* < 0.0001), highlighting a sharp rise in salinity, likely due to intensified saltwater intrusion. Finally, Kobadak showed a significant positive trend (*F* = 364.35, *p* < 0.0001), indicating a substantial increase over time, with a high *F*-ratio further affirming the robustness of the trend and suggesting considerable salinisation.

### Seasonal salinity variation

**Fig. 4:**
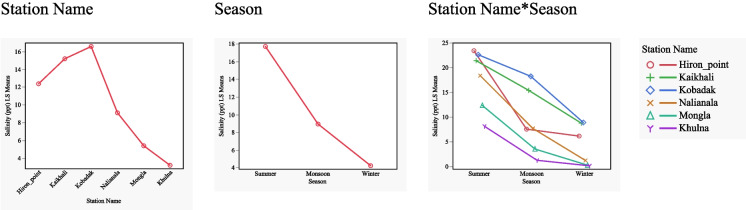
Seasonal (summer (Mar–Jun), wet monsoon (Jul–Oct), and dry winter (Nov–Feb)) variation in salinity (ppt) at six sites in the Sundarbans. The least squares means plots show the estimates of marginal means for station name, seasons, and their interactions

Understanding the seasonal salinity dynamics is important for managing biodiversity in the Sundarbans. We employed a two-way ANOVA to investigate spatiotemporal patterns in salinity across multiple monitoring stations and seasons in the three distinct seasons: summer (Mar–Jun), wet monsoon (Jul–Oct), and dry winter (Nov–Feb). The results revealed a highly significant effect of the seasons, between stations, and interaction between stations and seasons (*p* < 0.0001), meaning the magnitude of seasonal salinity change varies by station. Summer exhibited the highest mean salinity (17.724 ppt), followed by Monsoon (8.960 ppt) and Winter (4.254 ppt) (Fig. 4 and Table 2).

**Table 2:** Two-way ANOVA results of the effect of season (summer (Mar–Jun), monsoon (Jul–Oct), and winter (Nov–Feb)), station, and the interaction between station and season

Source	DF	L-R chi-square	Prob > ChiSq
Station name	5	1295	<.0001*
Season	2	1300	<.0001*
Station name*Season	10	1261	<.0001*

Figure 4 reveals a significant interaction between stations and seasons (*p* < 0.05), indicating that seasonal salinity differences were inconsistent across stations. Examination of the least squares means showed that the magnitude of seasonal variation differed substantially among stations. At Hiron Point, salinity decreased from 23.425 ppt in Summer to 6.163 ppt in Winter (a difference of 17.262 ppt), while at Kobadak, salinity decreased from 22.619 ppt in Summer to 8.905 ppt in Winter (a difference of 13.714 ppt). At Khulna, salinity decreased from 8.149 ppt in Summer to 0.154 ppt in Winter (a difference of 7.995 ppt). These differences in seasonal amplitude demonstrate that certain stations experience more pronounced seasonal fluctuations than others.

The station effect also varied by season, as evidenced by the interaction effect. During summer, the difference between stations of the highest (Hiron Point, 23.425 ppt) and lowest (Khulna, 8.149 ppt) average salinity was 15.276 ppt. In contrast, during winter, this difference was reduced to 8.751 ppt (between Kobadak, 8.905 ppt, and Khulna, 0.154 ppt). This reconfirms that spatial variability in salinity is most pronounced during the summer and less extreme during the winter.

**Fig. 5:**
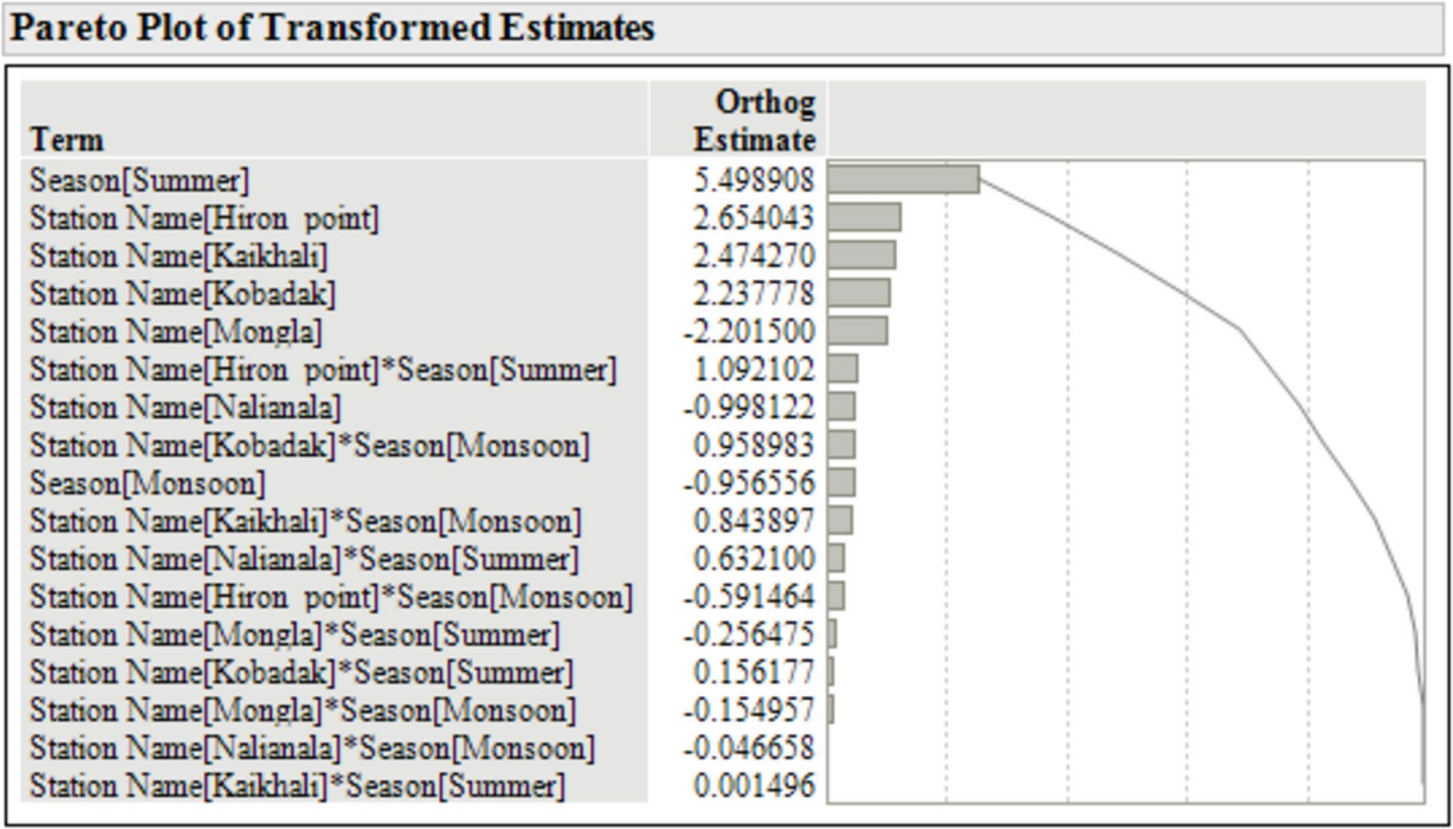
Pareto plot of standardised effect sizes for season, stations, and station*season effects in the two-way ANOVA of salinity

A Tukey’s honestly significant difference (HSD) post hoc test to evaluate pairwise differences among the Season*Station interaction assigned all groups the same letter (“A”), indicating no statistically significant differences in mean salinity across the season*station interaction. In other words, the Tukey procedure concludes that all interactions are statistically indistinguishable at the 0.05 significance level. Therefore, a Pareto plot is included to highlight patterns and trends to prevent overclaiming management significance Fig 5.

The Pareto plot ranks the standardised effect sizes (orthogonal estimates) for season, stations, and station*season effects of the two-way ANOVA output. The seasonal effect, particularly for summer, demonstrated the largest standardised effect (5.499), indicating its substantial influence on salinity variations. Significant variations in salinity were observed among the six monitoring stations. Mean salinity levels ranged from 16.591 ppt at Kobadak (highest) to 3.190 ppt at Khulna (lowest), with intermediate values at Kaikhali (15.210 ppt), Hiron Point (12.384 ppt), Nalianala (9.110 ppt), and Mongla (5.390 ppt). The effect screening analysis confirmed that location was a significant determinant of salinity, with several stations (Hiron Point, Kaikhali, Kobadak) showing standardised effects between 2.2 and 2.7.

### Climatic influence on salinity

**Fig. 6:**
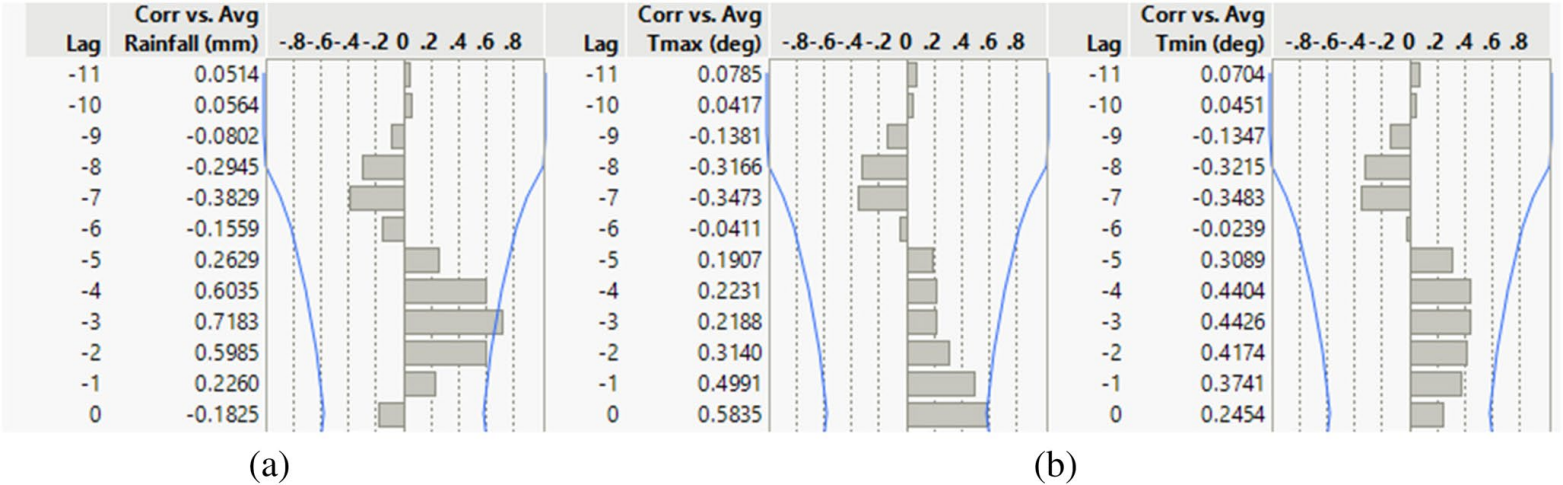
Temporal correlation between salinity and (**a**) rainfall and (**b**) temperature (*T*_max_ and *T*_min_) at Khulna. Lag is in months. Blue lines indicate 95% confidence intervals

**Fig. 7:**
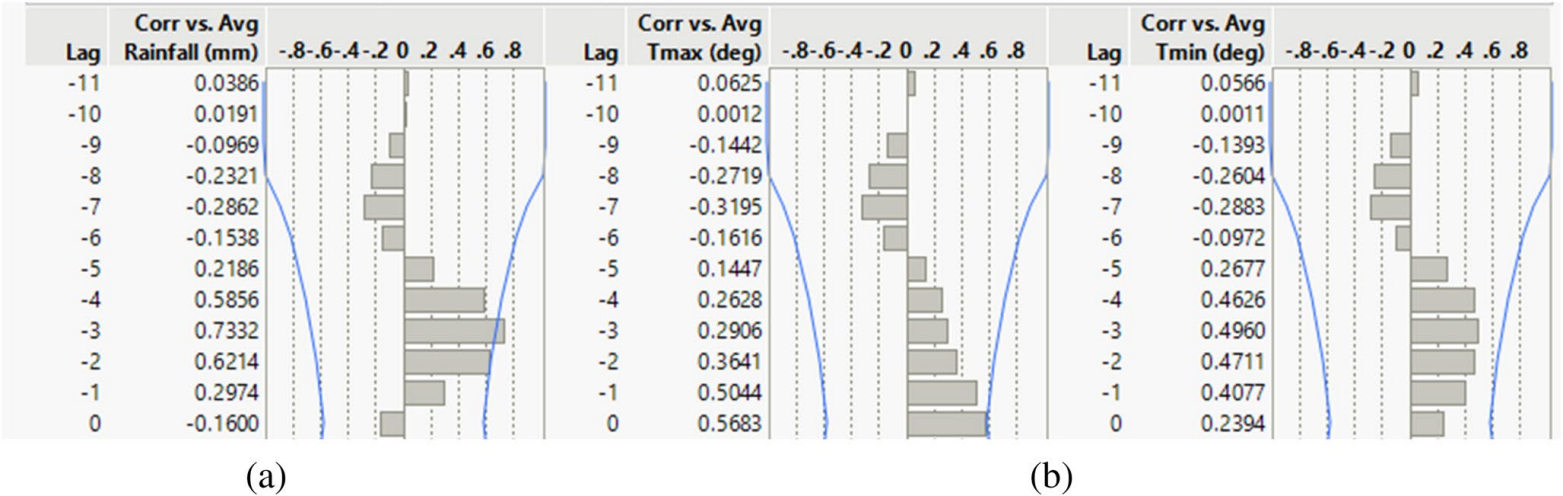
Temporal correlation between salinity and (**a**) rainfall and (**b**) temperature (*T*_max_ and *T*_min_) at Mongla. Lag is in months. Blue lines indicate 95% confidence intervals

We explored the climatic influence on salinity by correlating salinity versus rainfall and temperature for various monthly lags in rainfall and temperature. The correlations between the variables at Khulna and Mongla for the various lags are shown in Figs. 6 and 7.

**Fig. 8:**
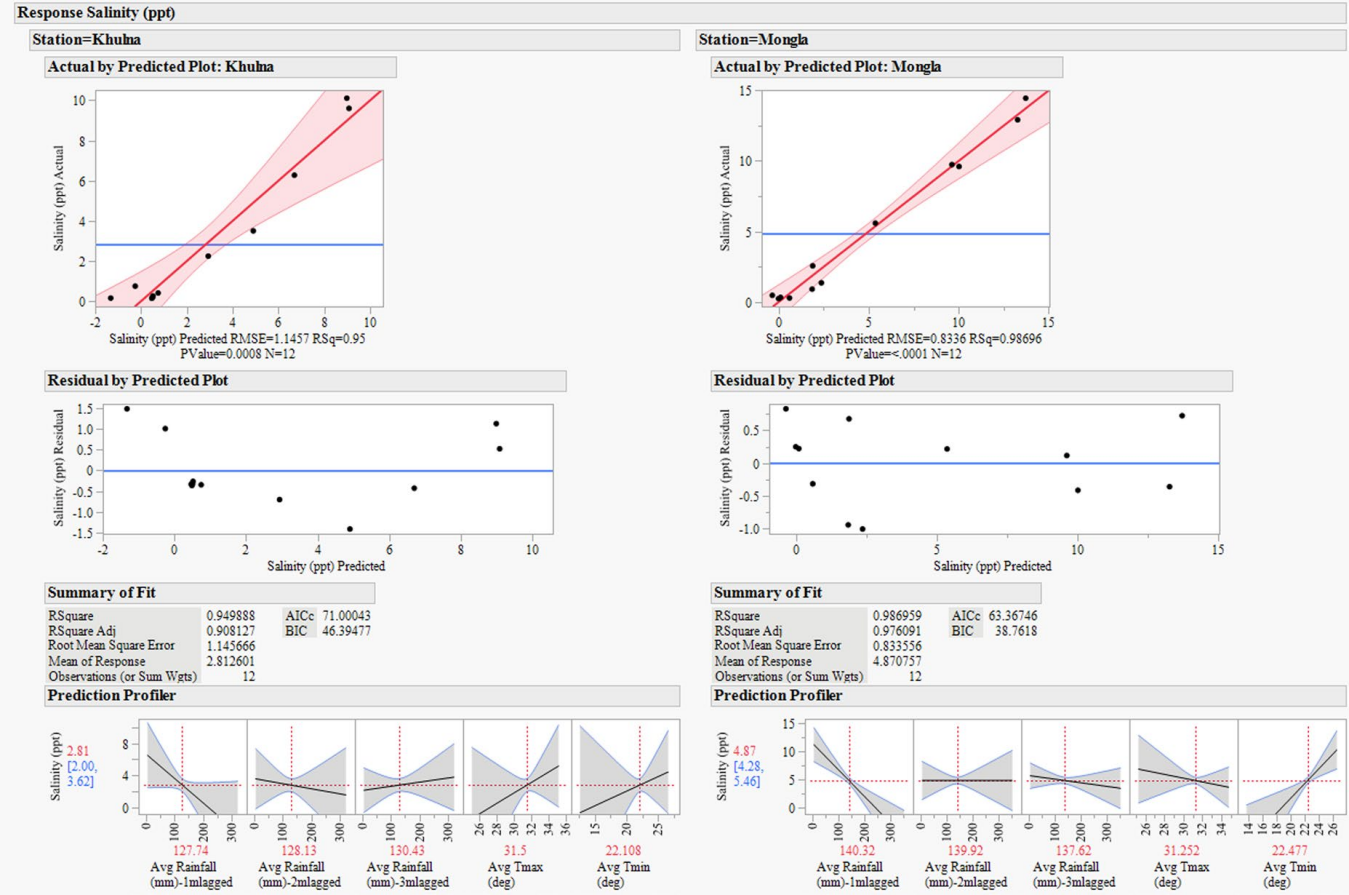
Multiple linear regression model diagnostics and prediction profiles for salinity at Khulna and Mongla using monthly lagged rainfall and temperature (2009–2022). The figure presents model performance metrics (Actual vs. Predicted plots, RMSE, *R*^2^, and *p*-values), effect summaries, residual diagnostics, and prediction profilers for salinity at Khulna and Mongla stations. Predictor variables include 1-, 2-, and 3-month lagged rainfall and both maximum and minimum temperature, highlighting the significance and direction of each climatic driver in explaining monthly salinity variability

The salinity versus rainfall three months before showed the highest correlation, above 0.7, which is moderately high and statistically significant. The salinity correlation against temperature is negligible. The results of the regression model are shown in Fig. 8.

The multiple regression model for salinity is based on five variables: 1-, 2-, and 3-month lagged rainfall, the monthly average maximum temperature, and the monthly average minimum temperature. We included variables that did not meet the significance threshold (*p* = 0.05). Our rationale for including predictors that are not individually significant is threefold. First, it helps to account for potential confounding influences and maintains the interpretability of lagged effects. Second, applying a consistent model structure at both stations allows for direct comparison of the influence and significance of each variable, enhancing the robustness of spatial inference. Third, temporal lag effects and interactions among climate variables may become significant with more detailed datasets. Excluding variables solely based on *p*-value risks omitting potentially important controls that are not currently significant but align with process understanding.

The regression models (Fig. 8) demonstrated strong predictive capacity for salinity at both stations, with high coefficients of determination. At Khulna station, the model achieved *R*^2^ = 0.95 (RMSE = 1.1457, *p* = 0.0008), while at Mongla station, the model performed marginally better with *R*^2^ = 0.98696 (RMSE = 0.8336, *p* < 0.0001). These metrics indicate that the selected predictor variables explain over 95% of the variance in salinity at both locations.

The prediction profiler (Fig. 8) indicates a negative relationship between lagged rainfall and salinity at both stations, confirming that increased rainfall leads to decreased salinity with a temporal delay of 1–2 months at Khulna and 1–3 months at Mongla. Temperature variables demonstrated more complex relationships, with minimum temperature showing a more decisive influence than maximum temperature, particularly at Mongla.

### Freshwater influence on salinity

**Fig. 9:**
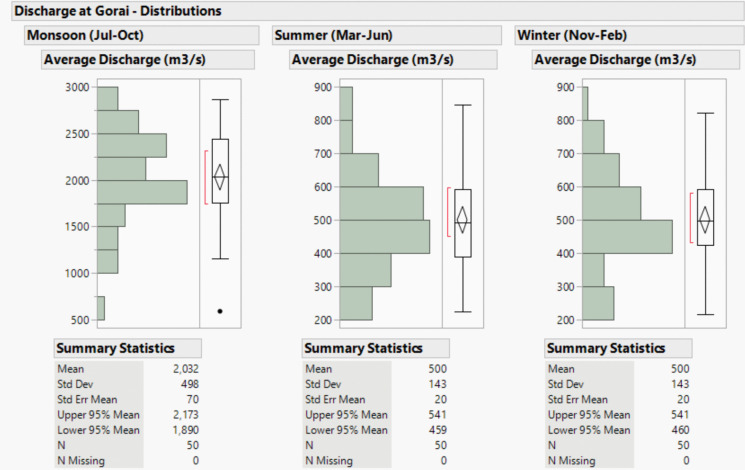
Discharge characteristics at Gorai Railway Bridge. The standard deviation indicates relatively lower variability compared to the monsoon season. The boxplots for summer and winter exhibit similar distributions, suggesting that discharge patterns are relatively stable outside the monsoon period

The river discharge during 1996–2022 at Gorai Railway Bridge (RB), where freshwater enters the upstream catchment (of the Sundarbans), shows a high degree of variability in absolute terms, as indicated by the high standard deviation (Fig. 9). The monsoon season experiences the highest discharge, with a mean flow of 2032 m^3^/s. The boxplot indicates a positively skewed distribution with a potential outlier at the lower end, suggesting occasional periods of exceptionally low discharge. The discharge reduces substantially during summer and winter.

We used the Mann–Kendall trend test to analyse whether there has been a noticeable increase or decrease in discharge at Gorai RB over time. The tests were done separately for the three seasons. The results suggest that monsoon discharge decreases with a slope of −0.0211 m^3^/s per day. An *R*-squared value of 0.282 indicated that the linear model explains about 28.2% of the variance in the trend component. However, the decreasing trends worsen in the summer (−0.0398 m^3^/s) and winter (−0.0468 m^3^/s) seasons. *p*-values of < 0.0001 indicate statistically significant trends. Other researchers have reported a decreasing trend in freshwater flow (Anwar & Takewaka, 2014; Islam & Gnauck, 2011), and our results statistically advanced previous findings, reporting separately for the seasons.

**Fig. 10:**
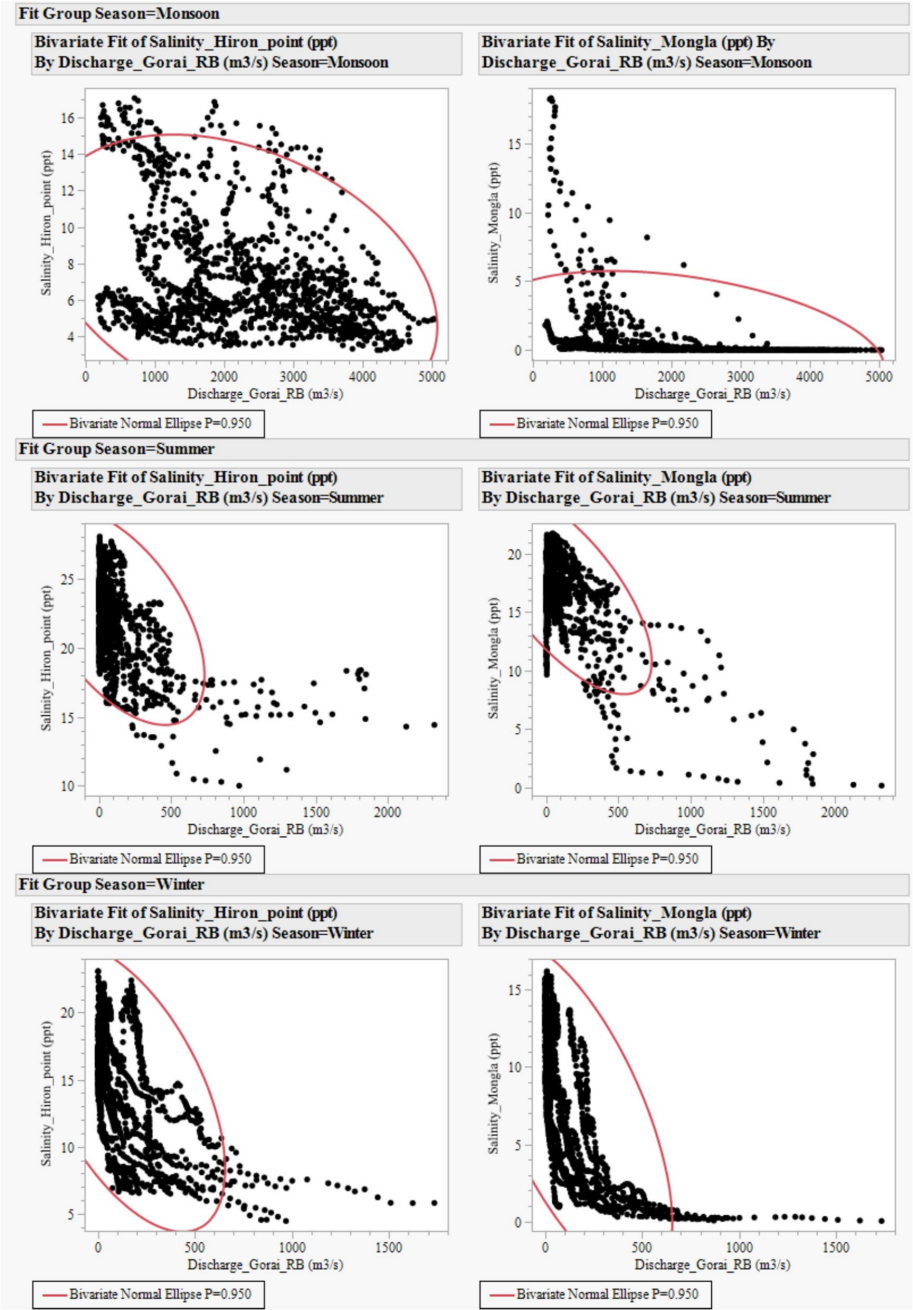
Discharge–salinity relationships in monsoon (Jul–Oct), summer (Mar–Jun) and winter (Nov–Feb) seasons. Panels display scatterplots of daily salinity (ppt) at Mongla and Hiron Point versus discharge at Gorai Railway Bridge (RB) (m^3^/s) during 2011–2022, stratified by season (monsoon, summer, winter). Each panel includes bivariate normal ellipse fits with 95% confidence intervals

The relationships between freshwater discharge at Gorai Railway Bridge (RB) and salinity levels at downstream stations such as Mongla and Hiron Point are complex and non-linear (Fig. 10). Spearman’s rank correlation analysis showed that discharge at the Gorai RB is inversely related to salinity at both Mongla and Hiron Point, with clear seasonal differences (Table 3). At Mongla, the relationship between discharge and salinity is particularly strong during the monsoon (*ρ* = −0.84) and winter (*ρ* = −0.78), highlighting the dominant role of freshwater inflow during these wetter periods. In contrast, summer correlations at Mongla are weaker (*ρ* = −0.23), indicating a greater influence of evaporation and tidal mixing when discharge is low.

**Table 3:** Seasonal Spearman’s rank correlation coefficients (*ρ*) between Gorai River discharge and salinity at Mongla and Hiron Point

Station	Season	Spearman’s *ρ*	*p*-value	Strength of correlation
Mongla	Monsoon	−0.84	< 0.0001	Very strong negative correlation
	Summer	−0.23	< 0.0001	Weak negative correlation
	Winter	−0.78	< 0.0001	Strong negative correlation
Hiron Point	Monsoon	−0.29	< 0.0001	Moderate negative correlation
	Summer	−0.46	< 0.0001	Moderate to strong negative correlation
	Winter	−0.54	< 0.0001	Strong negative correlation

Hiron Point showed moderate to strong negative correlations across all seasons, but these were generally weaker in magnitude compared to Mongla. The weakest correlation was during the monsoon (*ρ* = −0.29), possibly because of its closer coastal location, where salinity is also affected by marine water incursions and complex tidal mixing, especially during high discharge periods. Winter displayed the strongest correlation (*ρ* = −0.54), underlining discharge’s ongoing role in controlling salinity during colder months. These findings demonstrate that upstream discharge has a seasonal but mainly suppressive impact on salinity, which is vital for planning adaptive water management and salinity reduction strategies in the Sundarbans. Recognising the stronger link during monsoon and winter at Mongla helps identify key times when maintaining freshwater flows can most effectively lessen the impacts of saline intrusion.

We hypothesised that upstream freshwater inflow influenced salinity at different time lags in the Sundarbans. We employed the cross-correlation function (CCF) to assess the relationship between discharge at Gorai Railway Bridge (RB) and salinity levels at Mongla, as Mongla is the closest point to the Sundarbans ecosystem downstream of Gorai, and it is expected to respond most immediately. The Gorai flow does not directly affect salinity at the Kaikhali, Kobadak, and Nalianala stations, and there is no direct measurement of river discharge immediately upstream of these stations for cross-correlation analysis.

**Fig. 11:**
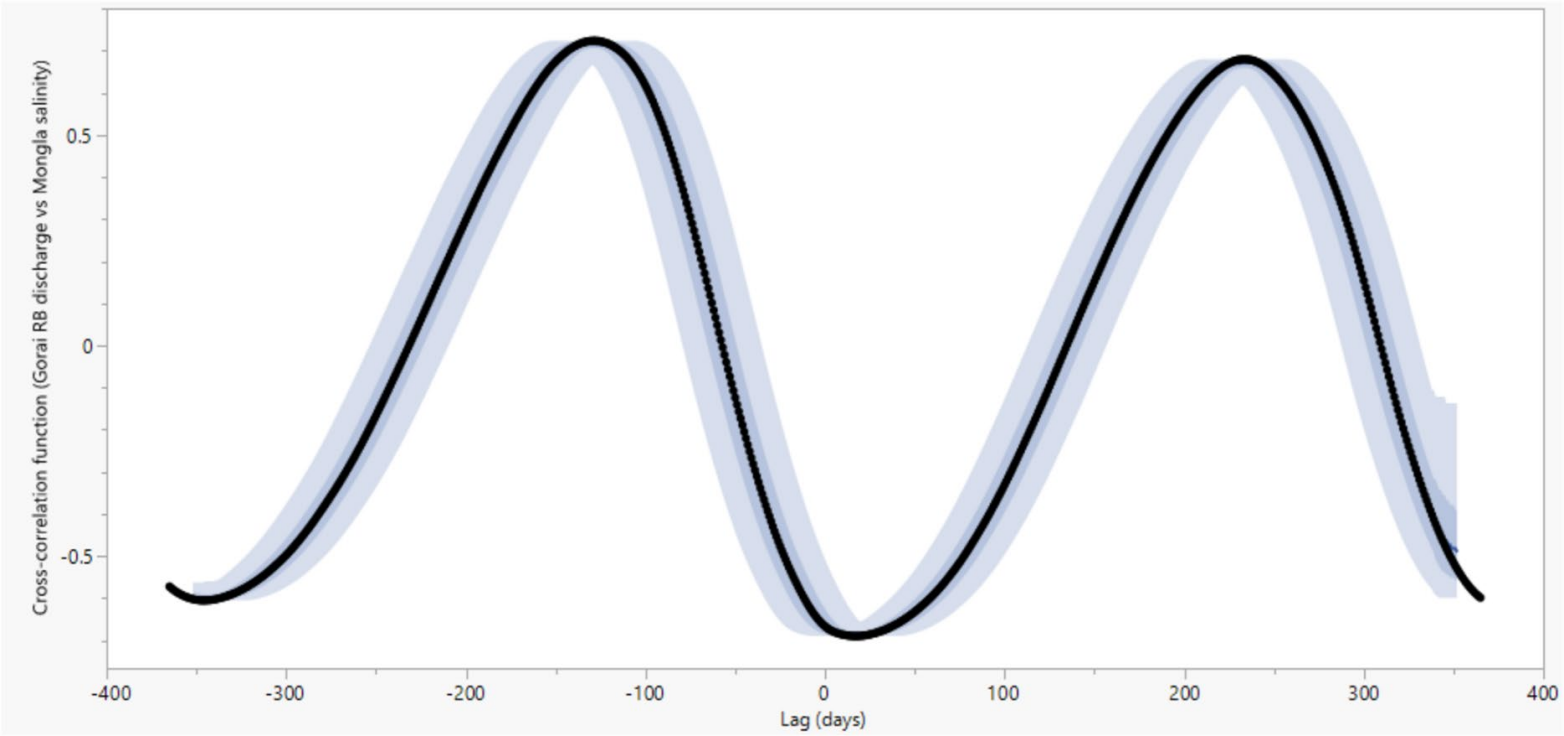
Cross-correlation between discharge at Gorai Railway Bridge (RB) and salinity at Mongla

The lag represents daily time steps and indicates the shift applied to salinity to calculate its correlation with discharge. For example, a lag of 10 means the salinity is shifted forward by ten days relative to the discharge data. We found a negative correlation (−0.65) between the discharge at Gorai RB and salinity at Mongla. Furthermore, we found that the upstream discharge at Gorai RB impacts salinity most at Mongla in the next 15 days (Fig. 11).

### Sea level influence on salinity

**Fig. 12:**
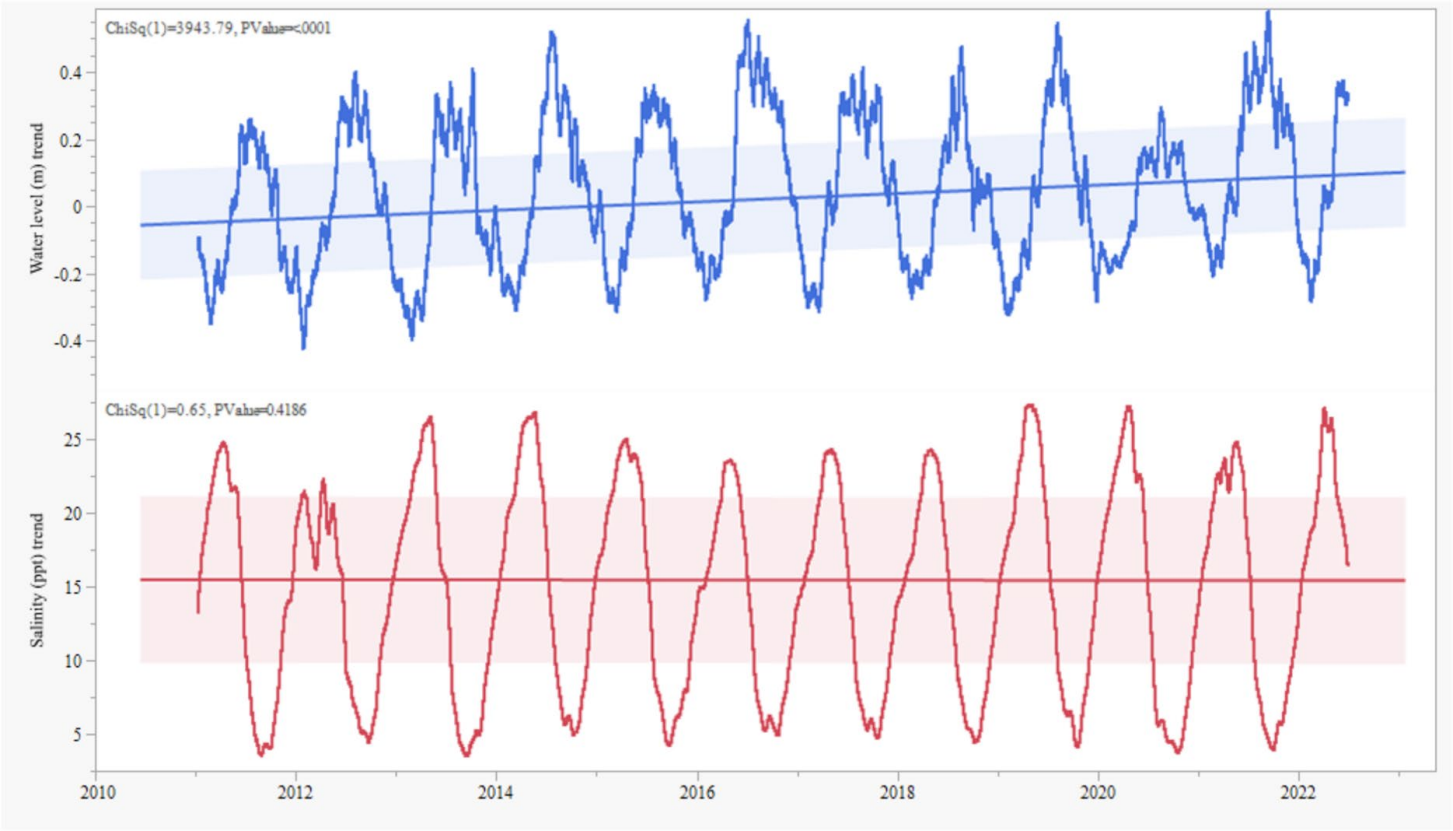
Water level and salinity trend near the seashore

Changes in tidal levels can influence salinity at the sea boundary. We used a statistical time series decomposition method to investigate the salinity and tidal level trends at Hiron Point. Time series decomposition splits the data into trend, seasonality, and residual components. Figure 12 presents the trend component from the analysis.

The trend component (Fig. 12) demonstrates that the water level trend at Hiron Point gradually increased between 2011 and 2022, possibly due to rising sea levels in the Bay of Bengal. Similar observations were made by Mondal et al. (2013). Their study revealed that the maximum high tidal levels are increasing at 18 mm per year, and the minimum low tidal water levels are decreasing at 8 mm per year at Khulna, 125 km inland from the Sundarbans coastline. At Hiron Point, the maximum high tide increased by 7 mm per annum, and the minimum low tide decreased by 4 mm per annum. Another researcher also documented an upward pattern at Hiron Point (Ashrafuzzaman et al., 2022). The salinity trend remained stable despite the increasing water level trend. The gradual rise in water levels at Hiron Point may not directly affect the salinity in the river; instead, factors such as rainfall and freshwater influx alongside oceanic mixing might contribute to a dilution of salinity near the seashore.

## Discussion

### Salinity variation and trends

The results provide robust evidence of both spatial and temporal intensification of salinity. They highlight the chronic exposure of seaward stations to high and rising salinity and the emerging risk of salinity intrusion into upstream areas. We found substantial differences in the central tendency and variability of salinity among stations. Inland stations like Kobadak and Kaikhali consistently exhibit wide interquartile ranges, indicating both chronic and episodic exposure to elevated salinity (Fig. 2). The trend analyses further elucidate these patterns by quantifying temporal changes in salinity at each station (Fig. 3).

In contrast, Khulna and Mongla salinities show much lower medians (0.41 and 2.90 ppt, respectively), with Khulna displaying a notably high outlier, suggesting occasional saline incursions into otherwise fresher environments. Earlier studies (Dasgupta et al., 2015; Rahman & Rahaman, 2018; Wahid et al., 2007) described the broad seaward-to-landward gradient of salinity in the Ganges-Brahmaputra-Meghna delta, but the present analysis provides a location-specific, quantitative assessment of both spatial heterogeneity and temporal evolution. The emergence of downward trends at upstream stations (Khulna, Mongla) suggests that the zone of saline influence has not migrated inland in the north-east side near the Sundarbans.

Our findings have critical ecological implications. The high and rising salinities at Kobadak, Kaikhali, and Hiron Point likely exceed the tolerance thresholds of many freshwater and brackish species (Chowdhury et al., 2016), threatening biodiversity and ecosystem services. For agriculture, the encroachment of salinity into areas like Nalianala portends increased vulnerability of rice and other staple crops (Mainuddin et al., 2021). However, we do not yet know the ecological and agricultural thresholds for salinity impacts at each station, limiting our ability to adopt targeted adaptation practices (e.g. salt-tolerant crops). We also do not know much about the effects of extreme events (e.g. cyclones) on the central tendency and outlier behaviour in salinity distributions.

### Seasonal salinity variation

The investigation showed that the Sundarbans exhibits significant seasonal differences, location-specific variations, and complex spatiotemporal interactions. Salinity differences between stations are most pronounced during summer. The high salinity levels observed during the summer season are consistent with findings from other studies in coastal and estuarine regions, where evaporation rates are typically highest, leading to increased salinity (Ranjan et al., 2006). The monsoon season brings a substantial influx of freshwater due to heavy rainfall, leading to a marked reduction in salinity across all stations. Following the monsoon, salinity levels begin to recover during the winter season, though they do not return to the peak levels observed in summer. This partial recovery can be attributed to reduced freshwater flow and increased tidal influences (Chowdhury et al., 2021). We note that the average winter salinity is lower than the monsoon (Fig. 4). This apparent contradiction stems from the averaging method used to summarise salinity across broad seasonal groupings. Since winter follows immediately after the monsoon, there can be a lag in salinity “recovery”—with freshwater from the just-completed monsoon still suppressing average levels (see Supplementary Materials for monthly mean salinity plots).

The significant spatial heterogeneity in salinity among stations likely reflects their relative positions within the estuarine continuum. Hiron Point exhibits the highest salinity during summer, sharply decreasing during the monsoon season. Kaikhali and Kobadak have a similar salinity pattern to Hiron Point, with peak salinity in summer. However, salinities at these locations do not drop in the monsoon like the Hiron point, as evidenced by their slope of the regression line. These stations are likely to be more exposed to saline water intrusion from the adjacent marine environment, a phenomenon well-documented in the literature (Chowdhury et al., 2023).

The spatial pattern of seasonal salinity variation extends the findings from past research (Hoque et al., 2006), which noted that stations closer to the Bay of Bengal experience more significant saltwater intrusion, particularly during winter. Conversely, Khulna, located further inland, shows the lowest overall salinity levels across all seasons, emphasising the protective effect of distance from the Bay of Bengal and the buffering capacity of freshwater inflows. The observed salinity levels at these stations are crucial for understanding the region’s potential impacts on agriculture, aquaculture, and drinking water resources.

The observed seasonal and spatial salinity patterns significantly affect water resource management in coastal regions. High salinity during the summer can lead to soil degradation, reduced agricultural productivity, and challenges for the drinking water supply around the Sundarbans. During the monsoon season, reduced salinity levels benefit fishing activity and agriculture in the adjoining areas but can lead to challenges in managing excess water and flooding. The partial recovery of salinity during winter suggests that this season may require particular attention in managing water resources to balance the needs of agriculture, fishing, and biodiversity conservation.

The Sundarbans salinity has highly pronounced seasonal and spatial gradients and a strong interaction, reinforcing the region’s need for season- and location-specific management strategies. The baseline understanding will help to produce seasonal location-specific outlook information. Adaptive management strategies, such as controlled targeted freshwater releases and salinity barrier installations, could be considered to mitigate the impacts of seasonal salinity variations (Feist et al., 2023).

### Climatic influence on salinity

The multiple linear regression analysis reveals that monthly salinity at Khulna and Mongla can be effectively predicted using lagged rainfall and temperature variables, with models explaining 95% and 99% of the variance, respectively. At Mongla, 1-month lagged rainfall (*p* = 0.002) and minimum temperature (*p* = 0.007) are highly significant predictors, indicating that recent rainfall and cooler temperatures play critical roles in modulating salinity. In contrast, at Khulna, 1-month lagged rainfall is the dominant predictor (*p* = 0.062), while temperature variables are not statistically significant. The 1-month to 3-month lag periods for rainfall to influence salinity indicate that while some rainfall reaches the estuary relatively quickly, the full impact of rainfall on salinity, especially in the context of the Sundarbans’ large and complex hydrological system, is manifested over a longer timeframe.

The negative relationship between lagged rainfall and salinity at both stations underscores the importance of freshwater input in controlling salinity intrusion. Residual diagnostics confirm the adequacy of the models, with no major violations of regression assumptions. The stronger model fit at Mongla suggests that this station is more sensitive to climatic variability, while additional hydrodynamic or anthropogenic factors may influence Khulna.

### Freshwater influence on salinity

The freshwater influence on salinity at Mongla and Hiron Point demonstrates a consistently strong and statistically significant negative correlation across all examined periods and seasons (all *p*-values < 0.0001). Higher discharges in the Gorai River are associated with marked reductions in salinity at both locations, with this effect persisting over the 2011–2022 period. The rate of salinity decline with increasing discharge appears to have moderated in recent years, as evidenced by a reduction in extreme salinity events at low flows, particularly at Hiron Point, which remains more sensitive to discharge variability than Mongla. Further research, potentially using numerical models, is needed to fine-tune the understanding of the freshwater discharge and salinity dynamics in these regions.

Seasonal analysis reveals pronounced differences, with winter periods (Fig. 4) consistently exhibiting the lowest salinity levels. At the same time, summer is characterised by the highest salinity, especially under low discharge conditions. These seasonal and spatial gradients underscore the critical role of riverine inflow and estuarine proximity in modulating salinity intrusion (Feist et al., 2023). In the Mekong Delta, upstream hydropower dam construction has been responsible for seawater penetrating more than 100 km inland (Huy et al., 2021; Thanh et al., 2023). Another study recorded a 45% rise, with a 30% extension of saltwater intrusion length (Eslami et al., 2019). Reports from Florida Bay describe higher salinities and extended saltwater intrusion following diversion events (Lorenz, 1999). These findings highlight the importance of sustained freshwater inputs in mitigating salinity intrusion and have significant implications for estuarine ecosystem health, agricultural productivity, and water resource management in the Ganges delta. The analysis of climatic and freshwater influences discussed here presents an opportunity to create a comprehensive predictive model utilising these factors in future studies.

### Sea level influence on salinity

Our simultaneous analysis of water level and salinity trends at the seafront, along with the partial decoupling of these two variables, reveals the presence of intense seasonal cycles, modestly rising water levels, and stable salinity during 2011–2022. However, the apparent decoupling of water level and salinity trends warrants further investigation. Despite a gradual increase in water level, salinity has remained stable on average, punctuated by intense seasonal cycles but lacking a clear upward or downward trend. If sustained, rising water levels could indicate increased vulnerability of the mangrove ecosystem. The decade-long stability of salinity is encouraging, but the high intra-annual variability still poses risks for biodiversity and ecosystem services.

## Conclusions

This study provides a detailed and nuanced understanding of salinity dynamics around the Bangladesh Sundarbans, revealing significant spatial heterogeneity and temporal trends across six monitoring stations. Our analysis shows that saline intrusion is progressively encroaching inland, as indicated by statistically significant upward trends in salinity at most coastal locations. These findings underscore the need to reassess management and conservation strategies in light of evolving hydrological and climatic conditions. Seasonal salinity patterns generally align with expectations, with peak salinity occurring during the dry summer months and the lowest average salinity found in winter rather than the monsoon season, a subtlety attributable to hydrological lag effects and tidal mixing.

There is a strong correlation between salinity, rainfall, and freshwater inflow over the previous three months. The relationship between freshwater inflow and salinity is complex and often non-linear, reinforcing the need for appropriate modelling approaches that capture such dynamics. The combined impacts of rising temperatures, variable precipitation patterns, and reduced freshwater inflows resulting from upstream interventions are likely accelerating salinity intrusion, with serious implications for the health of mangrove ecosystems and the livelihoods of the communities that depend on them. This study lays a foundation for targeted monitoring by elucidating station-specific vulnerabilities and the relative importance of climatic and hydrological controls. Despite the study’s attempts to utilise the best available data, significant gaps remain in the data and knowledge of the salinity dynamics of the Sundarbans, particularly in the numerous creeks that crisscross the forest. There is an urgent need for a comprehensive data monitoring and dedicated continuous assessment program to better understand and mitigate the impacts of climate change and development on the fragile Sundarbans coastal ecosystem.

## Supplementary Information

Below is the link to the electronic supplementary material.

ESM1 (DOCX.155 KB)

## Data Availability

No datasets were generated or analysed during the current study.

## References

[CR1] Akter, S., Wilson, C. A., Bhuiyan, A. H., Akhter, S. H., Steckler, M. S., & Rana, M. M. (2024a). Elevation dynamics between polders and the natural Sundarbans of the Ganges-Brahmaputra Delta Plain. *Estuaries and Coasts,**47*, 1877–1892. 10.1007/s12237-024-01349-4

[CR2] Akter, T., Hoque, M. A. A., Mukul, S. A., & Pradhan, B. (2024b). Coastal flood induced salinity intrusion risk assessment using a spatial multi-criteria approach in the south-western Bangladesh. *Earth Systems and Environment,**9*, 31–49. 10.1007/S41748-024-00399-9

[CR3] Anwar, M. S., & Takewaka, S. (2014). Analyses on phenological and morphological variations of mangrove forests along the southwest coast of Bangladesh. *Journal of Coastal Conservation,**18*(4), 339–357. 10.1007/s11852-014-0321-4

[CR4] Ashrafuzzaman, M., Santos, F. D., Dias, J. M., & Cerdà, A. (2022). Dynamics and causes of sea level rise in the coastal region of Southwest Bangladesh at global, regional, and local levels. *Journal of Marine Science and Engineering,**10*(6), Article 779. 10.3390/jmse10060779

[CR5] Aziz, A., & Paul, A. R. (2015). Bangladesh Sundarbans: Present status of the environment and biota. *Diversity,**7*(3), 242–269. 10.3390/d7030242

[CR6] Ball, M. C. (1988). Salinity tolerance in the mangroves *Aegiceras corniculatum* and *Avicennia marina*. I. Water use in relation to growth, carbon partitioning, and salt balance. *Functional Plant Biology,**15*(3), 447–464. 10.1071/PP9880447

[CR7] Bricheno, L. M., Wolf, J., & Sun, Y. (2021). Saline intrusion in the Ganges-Brahmaputra-Meghna megadelta. *Estuarine, Coastal and Shelf Science,**252*, Article 107246. 10.1016/J.ECSS.2021.107246

[CR8] Broucke, G., Nakamura, A., Osipova, E., & Wyatt, A. (2020). *Report of the joint WHC/IUCN Reactive Monitoring mission to The Sundarbans (Bangladesh)*.

[CR9] Chatterjee, M., Shankar, D., Vijith, V., Sen, G. K., Sundar, D., Michael, G. S., Amol, P., Chatterjee, A., Sanyal, P., Chatterjee, S., Basu, A., Chakraborti, S., Mishra, S. K., Suprit, K., Mukherjee, D., Mukherjee, A., Mukhopadhyay, S., Mondal, G., Kalla, A., & Das, M. (2021). Variation of salinity in the Sundarbans Estuarine System during the Equinoctial Spring tidal phase of March 2011. *Journal of Earth System Science,**130*(3), 1–25. 10.1007/S12040-021-01636-9

[CR10] Chowdhury, A., Naz, A., Bhattacharyya, S., & Sanyal, P. (2021). Dynamics of salinity intrusion in the surface and ground water of Sundarban Biosphere Reserve, India. *IOP Conference Series: Earth and Environmental Science,**944*(1), Article 012061. 10.1088/1755-1315/944/1/012061

[CR11] Chowdhury, A., Naz, A., Sharma, S. B., & Dasgupta, R. (2023). Changes in salinity, mangrove community ecology, and organic blue carbon stock in response to cyclones at Indian Sundarbans. *Life,**13*(7), Article 1539. 10.3390/LIFE13071539 37511914 10.3390/life13071539 PMC10381154

[CR12] Chowdhury, M. Q., De Ridder, M., & Beeckman, H. (2016). Climatic signals in tree rings of *Heritiera fomes* Buch.-Ham. in the Sundarbans, Bangladesh. *PLoS One,**11*(2), Article e0149788. 10.1371/journal.pone.0149788 26927229 10.1371/journal.pone.0149788 PMC4771160

[CR13] Chowdhury, M. Q., Sarker, S. K., Marma, M., Rahman, M. S., & Datta, A. (2024). Climate and salinity together control above ground carbon accumulation in the Sundarbans mangrove ecosystem. *Ocean and Coastal Management, 255, *107242-107242. 10.1016/j.ocecoaman.2024.107242

[CR14] Dasgupta, S., Hossain, M. M., Huq, M., & Wheeler, D. (2015). Climate change and soil salinity: The case of coastal Bangladesh. *Ambio,**44*(8), 815–826. 10.1007/s13280-015-0681-5 26152508 10.1007/s13280-015-0681-5 PMC4646857

[CR15] Dasgupta, S., Sobhan, I., & Wheeler, D. (2017). The impact of climate change and aquatic salinization on mangrove species in the Bangladesh Sundarbans. *Ambio,**46*(6), 680–694. 10.1007/s13280-017-0911-0 28470360 10.1007/s13280-017-0911-0 PMC5595742

[CR16] Eslami, S., Hoekstra, P., Nguyen Trung, N., Ahmed Kantoush, S., Van Binh, D., Duc Dung, D., Tran Quang, T., & van der Vegt, M. (2019). Tidal amplification and salt intrusion in the Mekong Delta driven by anthropogenic sediment starvation. *Scientific Reports*, *9*(1), 1–10. 10.1038/s41598-019-55018-9 10.1038/s41598-019-55018-9 PMC6904557 31822705

[CR17] Feist, S. E., Hoque, M. A., & Ahmed, K. M. (2023). Coastal salinity and water management practices in the Bengal Delta: A critical analysis to inform salinisation risk management strategies in Asian deltas. *Earth Systems and Environment,**7*(1), 171–187. 10.1007/S41748-022-00335-9

[CR18] Flowers, T. J., & Colmer, T. D. (2008). Salinity tolerance in halophytes. *New Phytologist,**179*(4), 945–963. 10.1111/J.1469-8137.2008.02531.X 18565144 10.1111/j.1469-8137.2008.02531.x

[CR19] Gain, A. K., & Giupponi, C. (2014). Impact of the Farakka Dam on thresholds of the hydrologic flow regime in the Lower Ganges River Basin (Bangladesh). *Water (Basel),**6*(8), 2501–2518. 10.3390/W6082501

[CR20] Ghosh, A., Schmidt, S., Fickert, T., & Nüsser, M. (2015). The Indian Sundarban mangrove forests: History, utilization, conservation strategies and local perception. *Diversity,**7*(2), 149–169. 10.3390/d7020149

[CR21] Ghosh, M. K., Kumar, L., & Roy, C. (2017). Climate variability and mangrove cover dynamics at species level in the Sundarbans, Bangladesh. *Sustainability,**9*(5), Article 805. 10.3390/su9050805

[CR22] Giri, C., Pengra, B., Zhu, Z., Singh, A., & Tieszen, L. L. (2007). Monitoring mangrove forest dynamics of the Sundarbans in Bangladesh and India using multi-temporal satellite data from 1973 to 2000. *Estuarine, Coastal and Shelf Science,**73*(1–2), 91–100. 10.1016/j.ecss.2006.12.019

[CR23] Gopal, B., & Chauhan, M. (2006). Biodiversity and its conservation in the Sundarban mangrove ecosystem. *Aquatic Sciences,**68*(3), 338–354. 10.1007/s00027-006-0868-8

[CR24] Haq, M. I., Shamsudduha, M., Zahid, A., Ahmed, K. M., Kamal, A. S. M. M., & Taylor, R. G. (2024). What drives changes in surface water salinity in coastal Bangladesh? *Frontiers in Water,**6*, Article 1220540. 10.3389/frwa.2024.1220540

[CR25] Hossain, M. I., Nabi, M. R., Ansari, M. N. A., Latif, A., MR, M., & Islam, M. S. (2016). Ecosystem Services of the World Largest Mangrove Forest Sundarban in Bangladesh. *International Journal of Innovation and Scientific Research*, *27*(1), 9–15. https://www.researchgate.net/profile/Md-Rejaun-Nabi-2/publication/331302242_Ecosystem_Services_of_the_World_Largest_Mangrove_Forest_Sundarban_in_Bangladesh/links/5c713fe8a6fdcc471595a566/Ecosystem-Services-of-the-World-Largest-Mangrove-Forest-Sundarban-in-Bangladesh.pdf

[CR26] Hossain, P. R. (2018). *Impacts of Climate Change on Coastal Ecosystems of Bangladesh* [Wageningen University]. 10.18174/443420

[CR28] Huy, B. L., Xuan, H. N., Van, N. T., & Le, H. H. (2021). The dangers of the construction of hydroelectric dams upstream of the Mekong River adversely effect on the ecosystems and livelihoods of people in the Mekong Delta, Viet Nam. *Environmental Challenges, 5, *100349-100349. 10.1016/j.envc.2021.100349

[CR29] Iftekhar, M. S., & Islam, M. R. (2004). Managing mangroves in Bangladesh: A strategy analysis. *Journal of Coastal Conservation,**10*(1), Article 139. 10.1652/1400-0350(2004)010[0139:mmibas]2.0.co;2

[CR30] Islam, S. N., & Gnauck, A. (2008). Mangrove wetland ecosystems in Ganges-Brahmaputra delta in Bangladesh. *Frontiers of Earth Science,**2*(4), 439–448. 10.1007/s11707-008-0049-2

[CR31] Islam, S. N., & Gnauck, A. (2011). Water salinity investigation in the Sundarbans rivers in Bangladesh. *International Journal of Water,**6*(1–2), 74–91. 10.1504/IJW.2011.043318

[CR32] Jabir, A. A., Hasan, G. M. J., & Anam, M. M. (2021). Correlation between temperature, sea level rise and land loss: An assessment along the Sundarbans coast. *Journal of King Saud University - Engineering Sciences*. 10.1016/j.jksues.2021.07.012

[CR33] Hoque, M. A., Sarkar, M. S. K. A., Khan, S. A. K. U., Moral, M. A. H., & Khurram, A. K. M. (2006). Present status of salinity rise in sundarbans area and its effect on sundari (Heritiera fomes) species. *Research Journal of Agriculture and Biological Sciences,**2*(3), 115–121.

[CR34] Khan, Z. H., Islam, M. S., Akhter, S., Hasib, M. R., Sutradhar, A., Timsina, J., Krupnik, T. J., & Schulthess, U. (2024). Can crop production intensification through irrigation be sustainable? An ex-ante impact study of the south-central coastal zone of Bangladesh. *PLOS Water,**3*(2), Article e0000153. 10.1371/journal.pwat.0000153

[CR35] Lorenz, J. J. (1999). The response of fishes to physicochemical changes in the mangroves of northeast Florida Bay. *Estuaries,**22*(2), 500–517.

[CR36] Mainuddin, M., Karim, F., Gaydon, D. S., & Kirby, J. M. (2021). Impact of climate change and management strategies on water and salt balance of the polders and islands in the Ganges delta. *Scientific Reports,**11*(1), 1–15. 33782450 10.1038/s41598-021-86206-1 PMC8007752

[CR37] Mirza, M. M. Q. (1998). Diversion of the Ganges water at Farakka and its effects on salinity in Bangladesh. *Environmental Management,**22*(5), 711–722. 10.1007/S002679900141 9680539 10.1007/s002679900141

[CR38] Mohiuddin, F. A. (2002). Effect of increased dredging length of the Gorai River off-take on lean season flow of the Gorai River in Bangladesh. In *Advances in Hydraulics and Water Engineering* (pp. 154–156). World Scientific Pub Co Pte Lt. 10.1142/9789812776969_0023

[CR39] Mondal, M. S., Jalal, M. R., Khan, M. S. A., Kumar, U., Rahman, R., & Huq, H. (2013). Hydro-meteorological trends in southwest coastal Bangladesh: Perspectives of climate change and human interventions. *American Journal of Climate Change,**02*(01), 62–70. 10.4236/ajcc.2013.21007

[CR40] Mukul, S. A., Alamgir, M., Sohel, M. S. I., Pert, P. L., Herbohn, J., Turton, S. M., Khan, M. S. I., Munim, S. A., Reza, A. H. M. A., & Laurance, W. F. (2019). Combined effects of climate change and sea-level rise project dramatic habitat loss of the globally endangered Bengal tiger in the Bangladesh Sundarbans. *Science of The Total Environment,**663*, 830–840. 10.1016/j.scitotenv.2019.01.383 30738263 10.1016/j.scitotenv.2019.01.383

[CR41] Qiu, C., & Wan, Y. (2013). Time series modeling and prediction of salinity in the Caloosahatchee River estuary. *Water Resources Research,**49*(9), 5804–5816. 10.1002/wrcr.20415

[CR42] Rahaman, S. M. B., Rahaman, M. S., Ghosh, A. K., Gain, D., Biswas, S. K., Sarder, L., Islam, S. S., & Sayeed, A. B. (2015). A spatial and seasonal pattern of water quality in the sundarbans river systems of Bangladesh. *Journal of Coastal Research*, *31*(2), 390–397. 10.2112/JCOASTRES-D-13-00115.1

[CR44] Rahman, M. M., & Rahaman, M. M. (2018). Impacts of Farakka barrage on hydrological flow of Ganges river and environment in Bangladesh. *Sustainable Water Resources Management,**4*(4), 767–780. 10.1007/s40899-017-0163-y

[CR45] Ranjan, P., Kazama, S., & Sawamoto, M. (2006). Effects of climate change on coastal fresh groundwater resources. *Global Environmental Change,**16*(4), 388–399. 10.1016/J.GLOENVCHA.2006.03.006

[CR46] Sarkar, I., & Chander, R. (2002). Co-seismic spring flow changes attributed to the March 29, 1999 Chamoli earthquake of Garhwal Himalaya. *Geosciences Journal,**6*(3), 181–186. 10.1007/BF02912688

[CR47] Sarkar, S. K., Ahmed, M.K., & Satpathy, K. K. (2019). Chapter 7 - The sundarban delta complex. In C. Sheppard (Ed.), *World Seas: An Environmental Evaluation* (Second). Academic Press. 10.1016/B978-0-08-100853-9.00009-9

[CR48] Snead, R. E. (2010). Bangladesh. In E.C.F. Bird (Ed.) *Encyclopedia of the World’s Coastal Landforms* (pp. 1077–1080). Springer Dordrecht. 10.1007/978-1-4020-8639-7_201

[CR49] Spalding, M., Kainuma, M., & Collins, L. (2010). World atlas of mangroves (1st ed.). *Routledge*. 10.4324/9781849776608

[CR50] Thanh, T. N., Huynh Van, H., Vo Minh, H., & Tri, V. P. D. (2023). Salinity intrusion trends under the impacts of upstream discharge and sea level rise along the Co Chien River and Hau River in the Vietnamese Mekong Delta. *Climate,**11*(3), Article 66. 10.3390/CLI11030066

[CR51] Trivedi, S., Zaman, S., Chaudhuri, T. R., Pramanick, P., Fazli, P., Amin, G., & Mitra, A. (2016). Inter-annual variation of salinity in Indian Sundarbans. *Indian Journal of Geo-Marine Sciences*, *45*(3), 410–415. https://www.wbnsou.ac.in/naac/journal_papers/54.%20GA-3.4.3-2016-01.pdf

[CR52] Uddin, Md Nazim and Haque, A. (2010). Salinity response in Southwest coastal region of Bangladesh due to hydraulic and hydrologic parameters. *International Journal of Sustainable Agricultural Technology*, *6*(3), 1–7. ISSN 1815-1272 https://vuir.vu.edu.au/id/eprint/32439

[CR53] Wahid, S. M., Babel, M. S., & Bhuiyan, A. R. (2007). Hydrologic monitoring and analysis in the Sundarbans mangrove ecosystem, Bangladesh. *Journal of Hydrology,**332*(3–4), 381–395. 10.1016/J.JHYDROL.2006.07.016

[CR54] Zaman, S., Bhattacharyya, S. B., Pramanick, P., Raha, A. K., Chakraborty, S., & Mitra, A. (2013). Rising water salinity: A threat to mangroves of Indian Sundarbans. *Community, Environment and Disaster Risk Management,**13*, 167–183. 10.1108/S2040-7262(2013)0000013014/FULL/XML

[CR55] Zhang, Z., Ahmed, M. R., Zhang, Q., Li, Y., & Li, Y. (2023). Monitoring of 35-year mangrove wetland change dynamics and agents in the Sundarbans using temporal consistency checking. *Remote Sensing,**15*(3), Article 625. 10.3390/rs15030625

