# Supplementary material for: Salinity dynamics in the Sundarbans of Bangladesh: influence of climate, freshwater inflow, and sea level changes
Source: Environ Monit Assess. 2025 Oct 20;197(11):1219. doi: 10.1007/s10661-025-14667-2 (PMC12537767; doi:10.1007/s10661-025-14667-2)
Supplement: Supplementary file 1 — (DOCX.155 KB) [file 10661_2025_14667_MOESM1_ESM.docx]

**Supplementary Information**

**Salinity Dynamics in the Sundarbans of Bangladesh: Influence of Climate, Freshwater Inflow and Sea Level Changes**

Shahriar Wahid^1,*^, Mohammed Mainuddin^2^, Francis Chiew^3^, Fazlul Karim^4^, Shaikh Nahiduzzaman^5^, Rubayat Alam^6^, Md. Raqubul Hasib^7^

^1^Commonwealth Scientific and Industrial Research Organisation (CSIRO), Canberra, Australia; Shahriar.Wahid@csiro.au

^2^Commonwealth Scientific and Industrial Research Organisation (CSIRO), Canberra, Australia; Mohammed.Mainuddin@csiro.au

^3^Commonwealth Scientific and Industrial Research Organisation (CSIRO), Canberra, Australia; Francis.Chiew@csiro.au

^4^Commonwealth Scientific and Industrial Research Organisation (CSIRO), Canberra, Australia; Fazlul.Karim@csiro.au

^5^Institute of Water Modelling, Dhaka, Bangladesh; snn@iwmbd.org

^6^Institute of Water Modelling, Dhaka, Bangladesh; rba@iwmbd.org

^7^Institute of Water Modelling, Dhaka, Bangladesh; rqb@iwmbd.org

*Corresponding author:

Shahriar Wahid, Ph.D.

Commonwealth Scientific and Industrial Research Organisation (CSIRO)

Canberra

Australia

T +61 2 6246 4155

Shahriar.Wahid@csiro.au

ORCID: 0000-0003-1117-4148


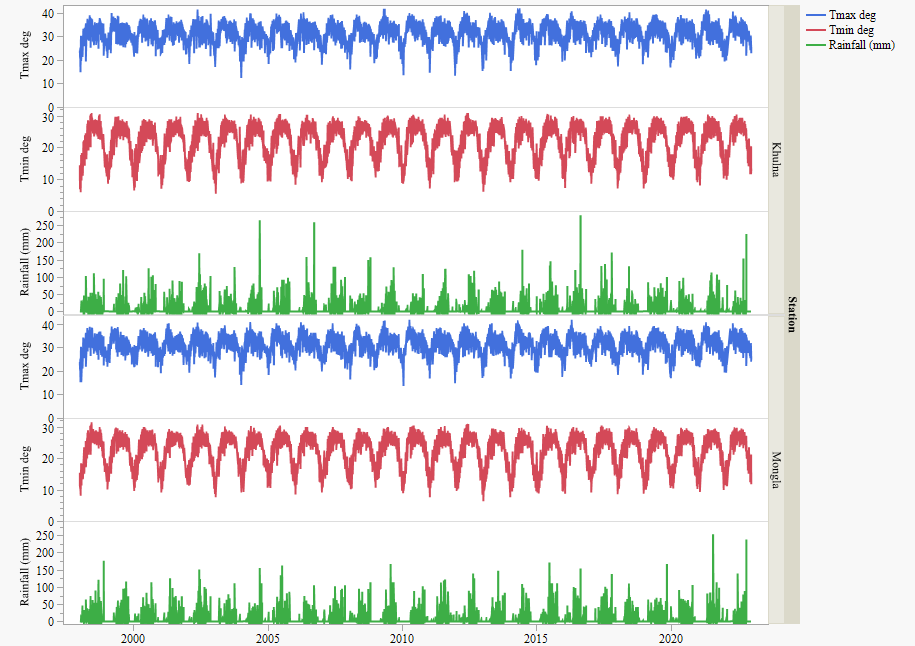


Fig. 1 Time-series plots of rainfall, maximum and minimum temperature at Khulna and Mongla stations


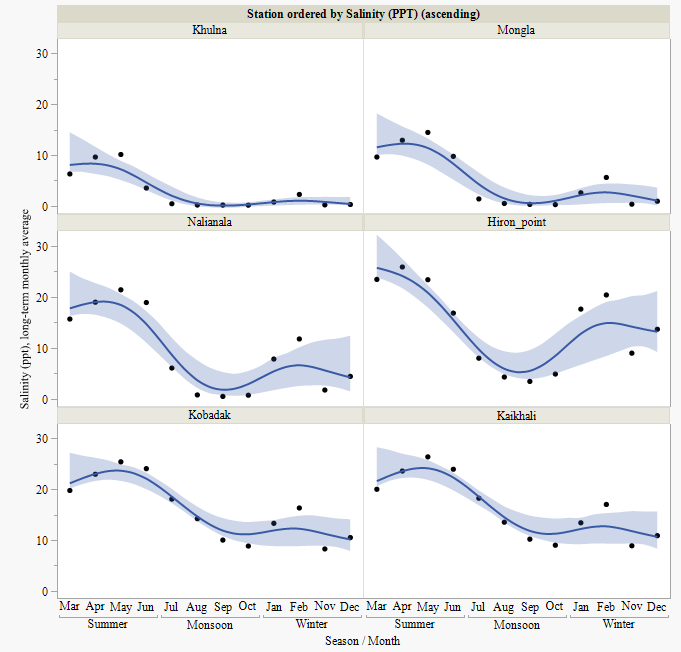


Fig. 2 Mean monthly salinity at Khulna and Mongla stations
